# Ecotypic divergence in Mongolian Scots pine persists via large-effect genetic adaptation and phenotypic plasticity

**DOI:** 10.1186/s12870-025-07695-0

**Published:** 2025-11-26

**Authors:** Wuyuntana, Qihui Fong, Chong-Yi Ke, De-Ming Gao, Min-Xin Luo, Yong-Zhi Yang, Run-Hong Gao, Pei-Chun Liao

**Affiliations:** 1 College of Forestry, Inner Mongolia Agricultural University, Huhhot, 010019 China; 2 Department of Life Science, National Taiwan Normal University, No. 88 Ting-Chow Rd., Sec. 4, Taipei, 116 Taiwan; 3 Department of Ecology and Evolutionary Biology, University of Michigan, Ann Arbor, MI 48109-1085 USA

**Keywords:** Ecological genomics, Ecotypic divergence, Genotype-environment interaction, Local adaptation, Mongolian Scots pine, Reference-free genomics

## Abstract

**Background:**

Understanding how ecotypic divergence persists under extensive gene flow is critical for predicting adaptive responses in long-lived conifers. Mongolian Scots pine (*Pinus sylvestris* var. *mongolica*) occupies contrasting mountain and sandy-dune habitats in northeastern China, forming two ecotypes with distinct environmental adaptations. Using reference-free Specific Locus Amplified Fragment sequencing (SLAF-seq) data and genome-wide SNPs, we explored patterns of genomic differentiation and genotype-environment associations to determine whether adaptive divergence is driven by few large-effect loci or polygenic shifts.

**Results:**

Despite weak population structure, we identified ~ 3% of the genome as adaptive outliers with strong differentiation between ecotypes. These loci exhibited significant environmental associations indicating that both adaptive genomic islands and polygenic shifts are dominated by large-effect variants rather than minor-effect alleles. Furthermore, phenotypic differentiation between ecotypes reflected a dual mechanism: adaptive genetic divergence shaping hydraulic traits and mechanical support, and genotype-by-environment (G×E) interactions enabling phenotypic plasticity in physiological responses, such as water-use efficiency and stress tolerance.

**Conclusion:**

The interplay between hard genetic adaptation and plasticity highlights how Mongolian Scots pine can simultaneously preserve ecotypic differentiation and respond flexibly to environmental heterogeneity. Importantly, such plasticity may provide a critical buffer against rapid climate change, allowing populations to persist despite ongoing gene flow and delayed genomic divergence. Our study highlights how divergent selection and plasticity together maintain ecotypic divergence in wind-pollinated pine variety with high gene flow and demonstrates a novel reference-free approach for dissecting adaptive architectures in non-model genomes.

**Supplementary Information:**

The online version contains supplementary material available at 10.1186/s12870-025-07695-0.

## Introduction

Dobzhansky’s [1] “Environment creates differences, and selection preserves them”, highlighted that spatially varying selection in heterogeneous environments differentiates traits, driving intraspecific divergence. Such a divergent selection maintains genetic polymorphisms despite ongoing gene flow [2, 3], particularly when multiple ecological niches exert different fitness optima on distinct genotypes [4]. These processes give rise to ecotypes—populations that are precisely adapted to their local environments, possessing heritable traits that enhance their fitness. As ecotypes accumulate genetic differences aligned with ecological variation, they can become the precursors of reproductive isolation and eventually ecological speciation [1, 5].

*Pinus sylvestris* var. *mongolica* (Pinaceae), commonly known as Mongolian Scots pine, is native to northeastern China and parts of Mongolia. It thrives in sandy soils, mountain ridges, and sunlit slopes at elevations between 400 and 900 m, demonstrating remarkable resilience to cold, drought, and nutrient-poor conditions, maintaining root function and growth under varying precipitation levels [6–8]. Its phenology is closely tied to the continental climate of its native range, characterized by frigid winters, hot summers, and significant diurnal temperature variations [9–12]. This resilience and ecological benefits make it a key species in afforestation efforts, particularly within China’s Three-North Shelterbelt Program, where it serves as a principal tree for windbreaks and sand stabilization in arid and semi-arid regions [6].

Mongolian Scots pine has two ecotypes: the mountain ecotype, found in the northern Greater Khingan Range (400–900 m a.s.l.), grows in rocky, well-drained soils on steep slopes, ridges, and sun-exposed aspects, typically in mixed stands with *Larix gmelinii* (Pinaceae) [13]. In contrast, the sandy-dune ecotype forms discontinuous, nearly pure forests on extremely nutrient-poor, mobile to semi-fixed sandy loams across the dune fields west and south of Hailaer [14]. The mountain ecotype may exhibit stronger cold-season growth limitation and earlier spring phenology [13], while the sandy-dune ecotype displays enhanced drought tolerance but reduced radial growth under low moisture [6, 14, 15]. These contrasts likely arise from local adaptation to contrasting water-energy regimes, genetic divergence, and differential phenotypic plasticity [16]. Major explanatory hypotheses include (1) adaptive genetic differentiation along moisture and temperature gradients [17] or on adaptive traits, like root architecture and water‐use efficiency [18], (2) divergent phenotypic plasticity in phenology and hydraulic regulation [6, 19], and (3) trade-off between growth and stress resistance [6]. To address these hypotheses, we can utilize *F*_ST_-outlier analysis and genotype-environment analysis (GEA) to investigate local adaptation through selection. For testing plasticity divergence, we can employ gene-by-environment (G×E) interaction mixed models. Additionally, comparing trait differences between ecotypes and quantifying their genetic (G), environmental (E), and G×E effects can provide insights into the trade-off between growth and stress resistance.

To evaluate adaptation versus plasticity hypotheses, we must examine how selection has shaped the genomes of different ecotypes. GEA tests and *F*_ST_-outlier scans can indicate whether alleles related to phenology, hydraulic traits, and growth-defence trade-offs cluster in a few differentiated regions or are spread across the genome. If selection targets a few large-effect genes, we will see high differentiation peaks against a uniform neutral background. Conversely, polygenic selection on numerous small-effect loci results in subtle, widespread shifts in allele frequencies, which may only be noticeable when analyzing adaptive SNPs collectively [20, 21]. Thus, comparing population structures based on (i) neutral SNPs and (ii) environmentally associated or high-*F*_ST_ SNPs helps determine if local adaptation drives the observed divergence between mountain and sandy ecotypes. Aligning adaptive clusters with ecotype identity indicates local adaptation. However, the lack of structure in both datasets suggests plasticity driven by environment-specific G×E responses [22].

When environmentally selected traits are controlled by multiple small-effect loci, the resulting divergence is predicted to form only shallow genomic islands that are hard to distinguish from the neutral genomic background [23]. The alternative “continents-of-divergence” model proposes that strong reproductive isolation arises when multiple adaptive polymorphisms become coupled through linkage disequilibrium (LD) and spread together across large chromosomal regions [24]. In conifers, however, LD typically decays within a few hundred base pairs because of very large effective population sizes and high recombination rates [25–27]. This rapid LD decay, combined with the high heterozygosity, extensive intraspecific diversity, outcrossing mating system, and efficient wind-mediated gene flow characteristic of pines [25], limits hitchhiking and makes the formation of large genomic continents unlikely. As a consequence, population structure inferred from neutral markers can appear weak or inconsistent with ecological boundaries, whereas analyses based on adaptive outlier loci reveal clear differentiation between mountain and sandy-dune ecotypes.

Distinguishing between adaptive divergence and phenotypic plasticity is challenging in non-model species like conifers, where genome-wide selection signatures can be subtle and LD decays rapidly. In conifers, determining LD is challenging due to their large genome sizes [28]. However, the gene effect on a specific environmental factor reflects the magnitude of the selection pressure and the possible range of influence on the linkage groups around the gene [24, 29]. This helps in the analysis of non-model species without reference genomes. Although *F*_ST_-outlier and GEA analyses provide indirect evidence of local adaptation, plastic responses to environmental variability can produce similar patterns. Thus, we integrate comparisons of environmental niches, GEA tests, and population structure analyses based on both neutral and potentially adaptive loci to clarify the genetic basis of ecotypic divergence.

In addressing the extent of genome-wide differentiation among these ecotypes, we specifically seek to answer the following questions: (1) How do these genetic patterns align with environmental and ecological divergence? (2) Does genomic divergence between ecotypes indicate local adaptation, or is it merely a result of phenotypic plasticity? (3) Are adaptive loci characterized by few large-effect genes or by polygenic adaptive shifts? Answering these questions is crucial for understanding the evolutionary mechanisms that maintain ecotypic variation in Mongolian Scots pine and for assessing its resilience to ongoing climate-driven challenges across its native range, which encompasses both cold, wind-exposed mountain ridges and arid, shifting sandy-dune systems.

## Materials and methods

### Sampling

**Fig. 1:**
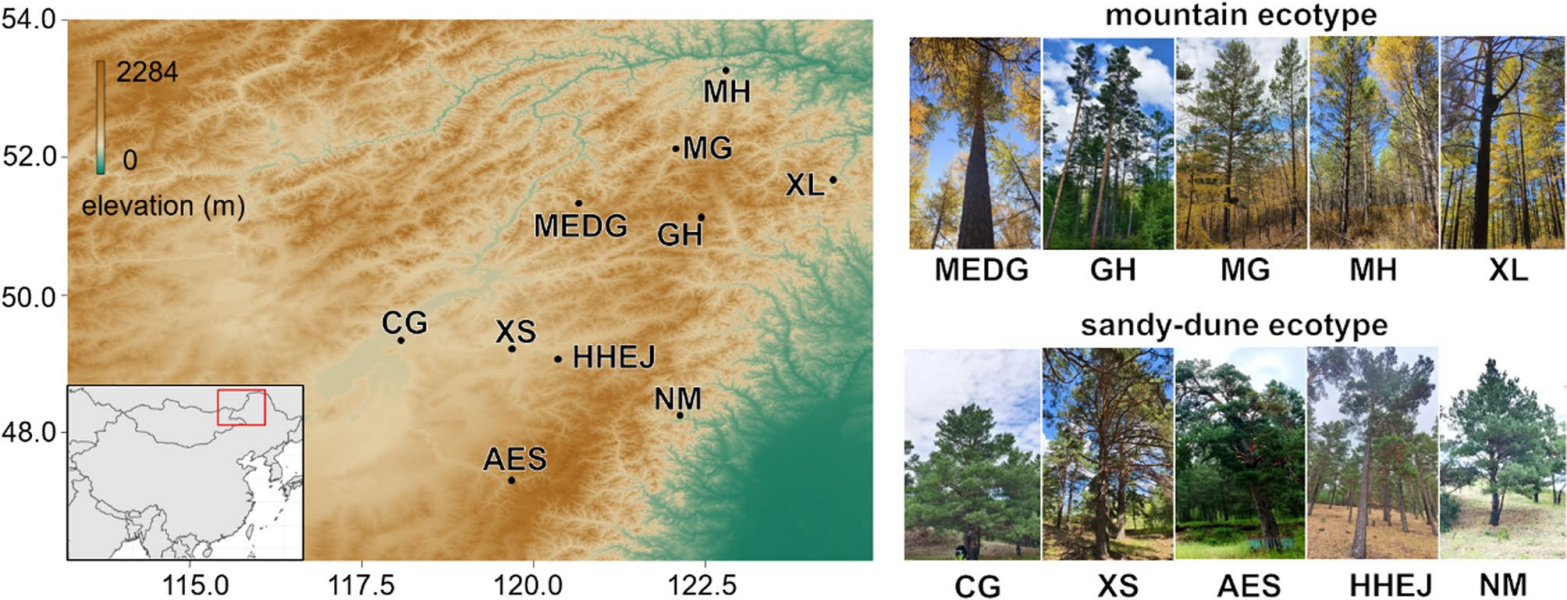
Sampling site and the growth form of two ecotypes. Ten natural populations (five populations per ecotype) were sampled in northeastern China for this study. The five top-right photos show the growth forms of each population in the mountain ecotype, while the five bottom-right photos depict the growth forms of each population in the sandy-dune ecotype

To determine if there is genetic differentiation between the mountain and sandy-dune ecotypes of Mongolian Scots pines, we sampled five populations from each ecotype in their natural distributions. The sampling covered the major natural distribution range of *P. sylvestris* var. *mongolica* in northeastern China, including both mountain and sandy-dune habitats. The sites were distributed across the northern Greater Khingan range and the Hailaer sand fields, representing the full known ecological and geographical extent of the variety (Fig. 1). Although some small, scattered marginal populations exist outside these regions, the sampled sites encompass the core distribution area and major ecotypes. We sampled 100 individuals of *P. sylvestris* var. *mongolica* to create a high-quality SNP dataset using the Specific Locus Amplified Fragment sequencing (SLAF-seq) reduced-representation approach. SLAF-seq is an optimized version of double digest restriction-site associated DNA sequencing. It can reduce sequence cost and avoid repetitive sequences with pre-designed reduced-representation library schemes [30]. Fresh current-year needles were collected from these populations (10 trees per natural population, spaced 50–100 m apart with similar diameters at breast height) and transported on ice to Biomarker Technologies (BMKGENE) in Beijing, China, for molecular analysis. All collections were performed with the permission of the relevant local forestry bureaus or forestry management authorities, in accordance with local regulations for plant material collection. No permit number was issued. The plant material was formally identified by Wuyuntana and Run-Hong Gao. The representative voucher specimen was collected and deposited in the Herbarium of Inner Mongolia Agricultural University (NMAC) under the accession numbers NMAC00023451–NMAC00023453.

### Genomic sequencing and variant calling

Total genomic DNA was isolated from the iced needle tissue following BMKGENE’s plant protocol. The concentrations and integrity of the DNA were confirmed using a Qubit and agarose electrophoresis prior to library construction. A double-digest SLAF design was optimized in silico, selecting the restriction enzymes HaeIII and Hpy166II to achieve even genomic coverage while minimizing repetitive fragments. Restriction fragments between 314 and 364 bp long were excised. Additionally, a rice cultivar, ‘Nipponbare’ (with a genome size of approximately 374 Mb), was digested and sequenced alongside the pine samples to verify enzyme performance and size selection. The pooled libraries were sequenced in rapid-run mode on an Illumina HiSeq 2500, generating 2 × 125 bp paired-end reads.

Raw reads were demultiplexed, quality-filtered (with Q30 ≥ 90%), and clustered at 95% identity, where each cluster represented a single SLAF tag, and the highest-depth read per tag was used as the provisional reference sequence. Clean reads from each individual were aligned to their respective tag-level reference using BWA-MEM (default parameters). SNPs were called independently with GATK v4 HaplotypeCaller [31] and bcftools v1.17 [32], and only the intersection of both callers was retained to reduce false positives.

The raw call set was filtered for site completeness (with INT >0.80), site missing rate >0.6, minor allele frequency (MAF >0.05), and retained biallelic sites, in accordance with community practices for population genomic datasets. After filtering, a high-confidence SNP matrix suitable for downstream population genomic and GEA analyses was obtained. We then calculated observed heterozygosity (*H*_*O*_), expected heterozygosity (*H*_*E*_), and inbreeding coefficient (*F*_IS_) at both the population and ecotype levels using the R package hierfstat [33].

### Collection of environmental factors

To characterize ecological divergence between the two Mongolian Scots pine ecotypes, we first collated 33 site-specific environmental predictors: 19 bioclimatic variables from CHELSA v2.1 [34], 10 edaphic attributes from SoilGrids 2.0 [35], and four topographic metrics (elevation data was downloaded from WorldClim2.1 [36], and we calculated slope, aspect, and roughness with elevation data by using QGIS). The above environmental factors are all grid data with a resolution of 1 km^2^. Geo-referenced occurrence records for each ecotype were assembled from provincial forest-inventory plots, herbarium vouchers from GBIF [37], and our own field GPS points (*n* = 12 for the mountain ecotype, *n* = 15 for the sandy-dune ecotype).

Environmental differentiation between mountain and sandy-dune ecotypes was assessed using a multivariate statistical approach. To reduce collinearity among the 33 site-specific environmental variables, we conducted a variance inflation factor (VIF) analysis and excluded variables with VIF > 10. Principal Component Analysis (PCA) was then performed to summarize environmental variation across sites. To test whether these two ecotypes occupy distinct environmental niches, we applied a multivariate analysis of variance (MANOVA) on the first three PCA axes, followed by pairwise comparisons using permutational MANOVA (PERMANOVA) with 999 permutations. Additionally, we performed two-sample *t*-tests for each environmental variable to evaluate univariate differences between mountain and sandy-dune ecotypes.

### Environmental niche comparison

Niche overlap analysis between the mountain and sandy-dune ecotypes was conducted using the R package ecospat [38]. After extracting variables using site coordinates, we performed a PCA to reduce dimensionality and construct a multivariate environmental space following the package manual. The environmental niche of each ecotype was then estimated as a kernel density of occurrence in this PCA-derived environmental space. Results were visualized using 2D kernel density grids plotted along the first two PCA axes.

We quantified niche overlap using Schoener’s *D* [39] and Hellinger’s *I* [40], which range from 0 (no overlap/complete divergence) to 1 (complete overlap/high similarity). This assessment is more effective with ecological factors than geographical space, as it considers climate availability and range analogies. Background tests were conducted to determine if the ecological niches of two ecotypes differ beyond what is expected based on their environmental requirements [39]. We also performed niche equivalency and niche similarity tests through randomization with 999 permutations. The equivalency test assesses whether the two ecotypes occupy different niches, while the similarity test checks if their niches are less alike than chance would suggest.

### Genotype-environment association study and population structure

Latent Factor Mixed Models (LFMM, with *K* = 2 latent factors) [41] and Redundancy Analysis (RDA) [42] identified SNPs that are significantly associated with the reduced environmental axes, and we reduced the false positive rate with the R package qvalue [43], candidate SNPs with an association *q*-value less than 0.05 are regarded as significantly associated with the environmental gradient. In addition to the *q*-value, we also intersect the results of both GEA methods to get robust environment-associated SNPs. Genome-wide *F*_ST_ outliers between two ecotypes were detected using fsthet [44], applying a percentile threshold of 99.5%. By intersecting these loci with the LFMM/RDA hits, we identified putatively adaptive variants whose allele frequencies are correlated with key climatic or edaphic gradients.

Additionally, we retained putatively neutral SNPs and *F*_ST_ outlier SNPs for population structure analysis. The sNMF [45] was run using settings for *K* ranging from 1 to 6, with 10 replicates for each value. The optimal *K* was determined using the Δ*K* method. PCA [46] was conducted separately on both sets to compare the neutral versus adaptive population structures. This integrated framework, which includes environmental-space comparison, GEA, and outlier tests, provides a robust basis for identifying genomic regions underlying ecotypic adaptation in Mongolian Scots pine.

Comparing the genetic structure of populations using neutral and adaptive SNPs helps us determine whether local adaptation or phenotypic plasticity drives divergence between ecotypes. If we observe weak or absent population structure with neutral loci, but clear differentiation is evident with adaptive loci, this pattern supports that local adaptation is the primary driver of divergence. In this case, selection has structured adaptive loci beyond the background of gene flow. Conversely, if neither the neutral nor the adaptive groups align with the ecotype categories, it suggests that the traits associated with the ecotypes arise mainly from phenotypic plasticity rather than fixed genetic differences.

### Determining the genetic pattern of adaptiveness

**Fig. 2:**
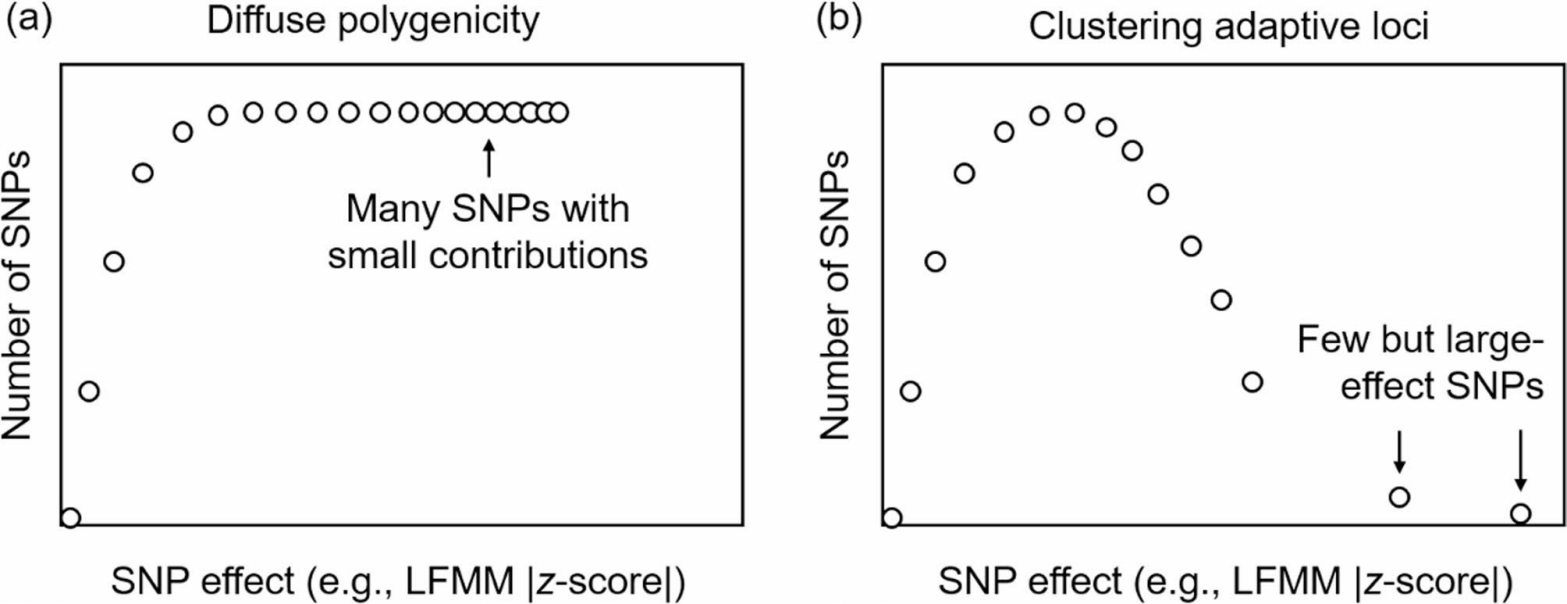
Schematic diagrams of effect-size spectra. **a** Multiple polygenic adaptive SNPs diffuse in the genome; (**b**) A few large-effect loci contribute to adaptive genomic islands

To determine the genetic architecture underlying adaptive divergence in response to environmental factors, we evaluated adaptive effect using the absolute LFMM *z*-score (|*z*|) and absolute RDA loadings from our analyses. We propose that if a few loci exhibit dominant effect, the resulting effect-size spectrum would reflect an oligogenic, long-tailed architecture. In contrast, if numerous loci contribute minor effects, the spectrum would display a polygenic, inverted L-shaped spectrum (Fig. 2).

### Gene-by-Environment interaction mixed models

To evaluate whether phenotypic plasticity in Mongolian Scots pine has a genetic basis and to disentangle it from local adaptation, we employed genome-wide G×E interaction mixed models using phenotypic and environmental data. To identify phenotypic traits potentially associated with ecotypic divergence, we first conducted two-sample *t*-tests comparing the mountain and sandy-dune ecotypes. Traits showing significant differences (*p* < 0.05) were retained for subsequent analysis. Phenotypic measurements were obtained from 10 individuals per population across five populations per ecotype (*n* = 50 per ecotype; total *n* = 100). For each individual, phenotypic traits were measured at both macroscopic and microscopic scales. Ten needle fascicles were randomly collected for external traits, including needle length (NL), needle width (NW), and needle thickness (NT), measured with a digital caliper (precision 0.01 mm). The mean of the 10 fascicles per individual was used for analysis. For anatomical traits, three fascicles per individual were sampled, and cross-sections were prepared from the mid-section of each needle to ensure consistency. Permanent transverse and epidermal sections were examined under a light microscope: at 100× total magnification (10× objective), we quantified xylem thickness (XT), phloem thickness (PT), conduction bundle width (BW), endodermis thickness (EnT), epidermis thickness (EpT), sclerenchyma thickness (ST), mesophyll thickness (MT), and resin canal number (RC); at 200× total magnification (20× objective), we measured stomatal length (SL), stomatal width (SW), and stomatal density (SD). For each individual, measurements from the three fascicles were averaged before subsequent analyses. Each test was conducted using individual-level trait data, and the results are summarized in Fig. S1.

Based on these results, we selected nine phenotypic traits related to needle and vascular tissue anatomy, including stomatal characteristics (density and width), conductive and supportive structures (xylem thickness, phloem thickness, sclerenchyma thickness, bundle width, and endodermis thickness), and needle size (length and thickness). These traits are closely associated with water regulation, gas exchange, mechanical support, and key physiological strategies for adapting to heterogeneous environments such as arid sandy plains and mountainous slopes. These traits provide an ecophysiological basis for investigating local adaptation and genomic divergence in Mongolian Scots pine (Table S1).

We modeled phenotypic variation using genetic (G), environmental (E), and genotype-by-environment (G×E) effects. The genetic component was represented by the first three principal components (PC1–PC3) derived from SNP-based PCA, capturing population-level genetic structure. The environmental component was derived from a separate PCA on environmental variables with high collinearity (VIF > 10), and the first two axes (Env_PC1 and Env_PC2), explaining over 50% of the total variance, were included in the model. These G and E components, along with their interaction (G×E), were included as fixed effects in the model, while population identity was modeled as a random effect to account for population structure. The model follows the formula:

$$Y_{ij}=G_i+E_j+{\left(G\times E\right)}_{ij}+\left(\left.1\right|\;Population\right)$$

where *Y*_*ij*_ is the trait value of the individual with genotype *i* in environment *j*, *Gi* is the genetic main effect, *E*_*j*_ is the environmental main effect, and (*G×E*)_*ij*_ represents the interaction term capturing phenotypic plasticity. A random intercept for population (1∣Population) was included to account for non-independence among individuals from the same population and to control for population structure.

Mixed-effects models were implemented using the lmerTest package in R (Kuznetsova et al.,2017), and significance was assessed using the anova() function. The Type III ANOVA was employed to evaluate the contribution of each main effect and interaction term, while controlling for the influence of all other terms in the model. For each trait, the sum of variance explained by PC1–PC3 was considered the genetic main effect (G), that by Env_PC1 and Env_PC2 as the environmental effect (E), and the cumulative variance explained by the interaction terms (i.e., PCs × Env_PCs) as the G×E interaction effect. These values were extracted from the ANOVA table associated with each model. Traits for which the genetic effect accounted for the largest proportion of variance were considered to reflect local adaptation, suggesting that genetic differentiation among populations corresponds to trait divergence. In contrast, traits primarily explained by environmental factors were interpreted as environmentally responsive, indicating phenotypic variation that aligns with environmental gradients, regardless of genetic background. Finally, traits with the greatest contribution from G×E interaction terms were inferred to exhibit phenotypic plasticity. That is, a context-dependent trait expression modulated by both genotype and environment, where different genotypes respond differently to environmental conditions. This pattern is consistent with a plastic response strategy that may buffer populations against environmental variability or stress.

### Gene function annotation

For the non-model-based variant calling method, we did not have the exact SNP positions. To determine which genes the environment-associated SNPs might be located on, we blasted the SLAF sequences against the target SNPs in the genus *Pinus* (taxon id: 3337) using the NCBI core nucleotide database. We retained blast results with an E-value of less than 0.05 and recorded the gene names from the accession descriptions. This framework enabled us to identify genetic variants associated with environmentally responsive traits and assess whether phenotypic divergence between ecotypes is primarily driven by fixed genetic differentiation (local adaptation) or genetically controlled plastic responses to habitat heterogeneity.

## Results

### Contrasting genetic structure between ecotypes inferred from neutral versus adaptive SNPs

After filtering, we generated a total of 279,267 SNPs. We retained 248,667 bi-allelic SNPs, which included 240,288 neutral SNPs and 8,379 positive outlier SNPs identified through fsthet analysis to differentiate between the two ecotypes. We then calculated population-based and ecotype-based observed heterozygosity (*H*_*O*_), expected heterozygosity (*H*_*E*_), and inbreeding coefficient (*F*_IS_) using the full bi-allelic dataset (Table 1).In all populations and ecotypes, *H*_*O*_ is lower than *H*_*E*_, resulting in positive *F*_IS_ values. Although pine trees are typically highly outcrossing due to wind pollination, elevated *F*_IS_ values in our dataset likely reflect a combination of biological and technical factors. On the biological side, Mongolian Scots pine is a geographically restricted variety of *P. sylvestris*, mainly distributed in northeastern China, where populations are relatively fragmented and effective population sizes are lower than those of the widespread Eurasian Scots pine. This is consistent with the overall lower heterozygosity (*Ho*) observed in our dataset compared to the Eurasian Scots pine [47]. On the technical side, our use of a reference-free, tag-level mapping strategy combined with filtering to retain only bi-allelic SNPs may lead to allele dropout and mapping bias, both of which can inflate *F*_IS_ estimates [48]. We therefore interpret these *F*_IS_ values cautiously, as they may partially reflect reduced genetic diversity and mating connectivity in this variety, rather than true inbreeding in the classical sense.

**Table 1:** Population-based and ecotype-based estimates of observed heterozygosity (*H*_*O*_), expected heterozygosity (*H*_*E*_), and inbreeding coefficient (*F*_IS_)

Population	Code	*H*_O_	*H*_E_	*F*_IS_†
Mountain ecotype		0.120	0.183	0.345
Man Gui	MG	0.108	0.180	0.313
Mohe	MH	0.113	0.179	0.296
Nanmu	NM	0.117	0.180	0.284
Xinlin	XL	0.123	0.180	0.252
Xishan	XS	0.120	0.177	0.262
Sandy-dune ecotype		0.121	0.183	0.344
Aershan	AES	0.112	0.175	0.285
Cuogang	CG	0.118	0.177	0.273
Genhe	GH	0.127	0.182	0.247
Honghuaerji	HHEJ	0.118	0.177	0.253
Moerdaoga	MEDG	0.114	0.181	0.297

†*F*_IS_ values should be interpreted cautiously due to the potential combined effects of limited population connectivity and technical biases associated with the reference-free mapping strategy

**Fig. 3:**
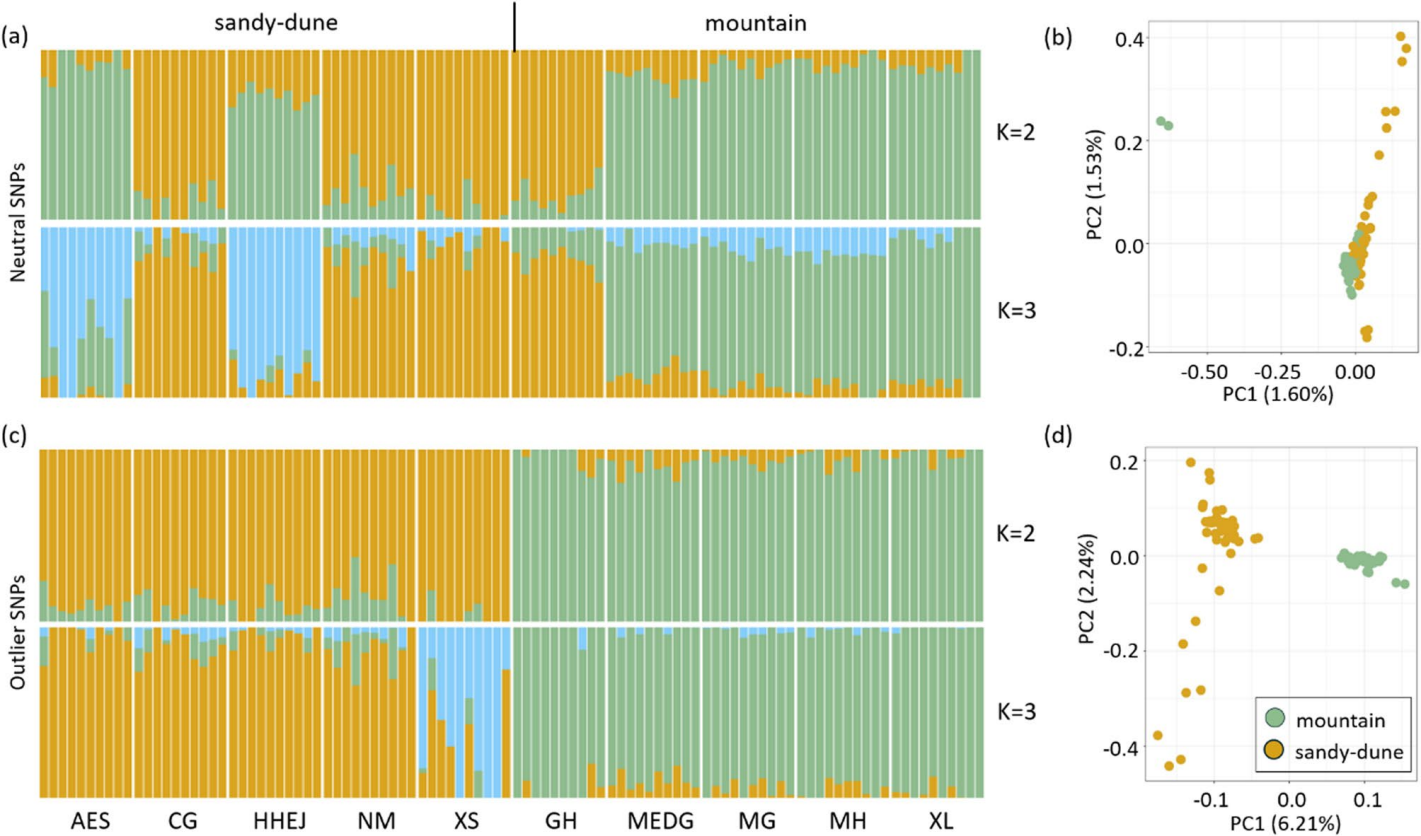
Genetic structure of neutral SNPs and *F*_ST_-outlier SNPs. In both datasets, the optimal number of genetic clusters (*K*) is 2. **a** The neutral genetic structure revealed by the sNMF analysis shows evidence of admixture, with the distribution of genetic components not corresponding to the ecotypes. When *K* = 2, AES, HHEJ, and GH exhibit distinct patterns. At *K* = 3, AES and HHEJ show a unique genetic component not shared with other populations. **b** A similar pattern is observed in the PCA based on neutral SNPs, further supporting the sNMF results. **c** The genetic structure based on *F*_ST_-outlier SNPs corresponds closely to the ecotypes. At *K* = 3, XS displays a distinct genetic structure. **d** The PCA based on outlier SNPs reveals two clear clusters that group according to ecotype

Population genetic structure analysis by sNMF and PCA revealed incongruent genetic differentiation between the mountain and sandy-dune ecotypes for the neutral SNPs (Fig. 3). In this analysis, the AES and HHEJ populations of the sandy-dune ecotype clustered with the mountain ecotype, while the GH population from the mountain ecotype was associated with the sandy-dune ecotype (Fig. 3a). However, when the number of clusters (*K*) exceeded 3, the AES and HHEJ populations exhibited distinct major genetic components that diverged from the other populations, indicating local divergence despite their similarity to the mountain ecotype (Fig. S2a).

In contrast to the inconsistent clustering observed with neutral SNPs, the putative adaptive SNPs identified as positive outliers clearly distinguished the two ecotypes in the SNMF and PCA analyses (Fig. 3c and d). When *K* > 2, the XS population of the sandy-dune ecotype displayed a significantly different genetic clustering, suggesting potential local adaptation (Fig. S3a). This divergence was also evident in the PCA, where the XS population was distinctly separated from the main cluster of the sandy-dune ecotype on the PC2 axis (Fig. S3c).

Notably, two samples from the XL population (mountain ecotype) exhibited a very distinct genetic composition in both neutral and outlier SNPs when *K* >3 (Fig. S2, Fig. S3). This divergence may be due to genetic introgression from other species such as the closely related *P. densiflora*, which is naturally distributed in northeastern China. Previous studies have suggested that *P. sylvestris* var. *mongolica* and *P. densiflora* may have experienced secondary contact and hybridization in Northeast China, potentially giving rise to novel lineages (e.g., *P. funebris* and *P. takahasii*) [49]. Therefore, introgression from *P. densiflora* could explain the distinct genetic composition of these individuals. Moreover, interspecific hybridization within the genus *Pinus* has been repeatedly documented [50, 51], indicating that gene flow across species boundaries is possible, though further genomic analyses are required to verify this case.

### Environmental differentiation between mountain and sandy-dune ecotypes

After reducing multicollinearity among 33 site-specific environmental variables, we retained 13 variables with VIF < 10 (Table S2). These included three bioclimatic variables related to temperature and precipitation: the mean temperature of the coldest quarter (bio11, VIF = 1.077), annual precipitation (bio12, VIF = 1.104), and precipitation of the driest month (bio14, VIF = 1.054). In addition, six edaphic variables were retained, including cation exchange capacity (CEC, VIF = 1.529), volumetric fraction of coarse fragments (CFVO, VIF = 4.091), soil pH (pHH_2_O, VIF = 4.296), soil organic carbon content (SOC, VIF = 1.756), and the proportions of clay (VIF = 1.681) and silt (VIF = 1.447). All four topographic variables exhibited VIF < 10, indicating they were suitable for inclusion in subsequent analyses. These variables included aspect (VIF = 1.528), elevation (VIF = 1.465), roughness (VIF = 3.786), and slope (VIF = 4.246).

PCA of the 13 retained variables revealed that the first two axes, Env_PC1 and Env_PC2, explained 34.60% and 22.97% of the total variation, respectively. They together explained over 57.57% of the environmental variation (Table S3). Therefore, these two axes were deemed sufficient to represent environmental differences between the mountain and sandy-dune ecotypes.

A multivariate analysis of variance (MANOVA) conducted using the first two PCA axes indicated a marginally significant difference in environmental conditions between the ecotypes (Pillai’s trace = 0.676, *F* = 4.179, *p* = 0.065; Table S4). Similarly, a PERMANOVA based on Euclidean distances showed a comparable trend (*R*² = 0.214, *F* = 2.179, *p* = 0.068; Table S5), suggesting potential but not statistically significant environmental differentiation between the two ecotypes.

While both MANOVA and PERMANOVA indicated only marginal differentiation, individual two-sample *t*-tests identified several specific environmental variables that differed significantly between ecotypes (Fig. S4). For example, the proportion of clay in the soil showed a significant difference (*p* < 0.05), while both aspect and soil pH exhibited stronger differentiation (*p* < 0.01). The mean temperature of the coldest quarter (bio11) and the proportion of silt displayed the most significant differences (*p* < 0.001). These findings align with expectations, as latitude and soil composition represent the most pronounced environmental contrasts between the mountain and sandy-dune habitats.

### Detection of SNPs associated with environmental variables across ecotypes

For the GEA analyses, we employed two strategies. The first strategy involved using all 248,667 bi-allelic SNPs to identify genes generally associated with local environments. The second strategy focused on identifying environmentally associated genes that may be positively selected in diverging ecotypes using 8,379 outlier SNPs. In the first strategy, the LFMM and RDA detected 1,003 and 1,761 environment-associated SNPs, respectively, with an overlap of 127 SNPs. In the second strategy, we identified 150 SNPs with LFMM and 408 SNPs with RDA, resulting in an intersection of 46 SNPs (Table S6, Fig. S5). These 46 overlapping SNPs, identified by both LFMM and RDA, were primarily associated with slope and roughness, suggesting their potential relevance in edaphic adaptation between the ecotypes. We further blasted these environment-associated SNPs against the genus *Pinus* (taxon ID: 3337) in the NCBI core nucleotide database. Our results revealed that some SNPs are located in the genes *KOR1*, *SuSy1*, *Lac8*, *MYB8*, *CesA1*, and *CCoAOMT* (Table S7). These genes are typically involved in vascular bundle formation, a process related to plant growth and drought stress. Interestingly, the blast results may help explain the variations in vascular bundle phenotypes observed between ecotypes (Table S1).

### The genetic pattern of adaptiveness

**Fig. 4:**
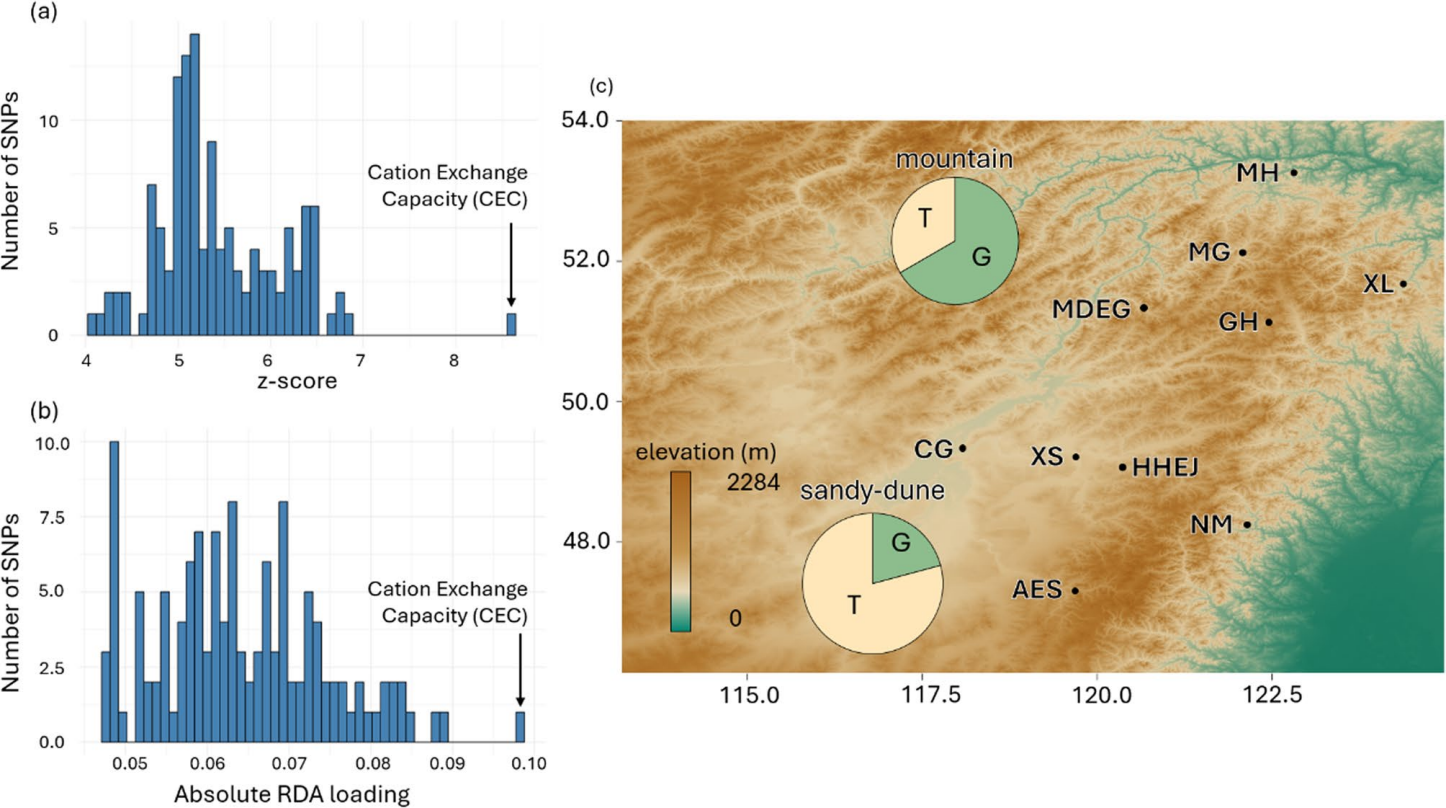
Effect-size spectra and allele frequencies of the SNP with the largest effect. **a** The effect-size spectrum based on absolute LFMM *z*-scores (|*z*|) and (**b**) the effect-size spectrum based on absolute RDA loadings. Both show a similar pattern that a small number of loci exhibit significantly large effects. **c** The SNP with the largest effect is associated with Cation Exchange Capacity (CEC), and its allele frequency differs markedly between ecotypes

We used absolute LFMM z-score (|*z*|) and absolute RDA loadings to evaluate the contribution of individual SNPs to adaptation. Our analysis revealed that |*z*| values of all 127 environment-associated SNPs exceeded 4, indicating that these adaptive SNPs all had large effects on environmental responses. Additionally, when examining the effect-size spectrum, we observed that although all SNPs exhibited a large effect, some had a dominant effect, resulting in an oligogenic, long-tailed curve (Fig. 4a and b).

Next, we focused on the most dominant SNP, which was associated with cation exchange capacity (CEC). We found that its allele frequency (G/T) varied between ecotypes. In the mountain ecotype, allele frequency of G was 0.667, while in the sandy-dune ecotype, allele frequency of G was 0.208 (Fig. 4c).

### Traits exhibit distinct genetic, environmental, and G×E interaction patterns

Based on the genotype-by-environment (G×E) interaction model, traits exhibited varying degrees of sensitivity to genetic (G), environmental (E), and G×E effects (Table 2). Traits predominantly influenced by genetic factors included stomatal width (SW, 46.38%) and xylem thickness (XT, 53.16%), both of which are associated with water-use strategies such as stomatal regulation and hydraulic conductance. These findings suggest that local adaptation may play a central role in shaping the structural basis for water management in plants. In contrast, traits primarily affected by the environmental component included needle-related morphological traits, such as needle length (NL, 54.31%) and needle thickness (NT, 70.23%), as well as phloem thickness (PT, 45.20%), which is associated with nutrient transport. These environmentally sensitive traits indicate that the foliar morphology of Mongolian Scots pine is highly plastic and responsive to local habitat conditions. Lastly, several traits were most strongly affected by G×E interactions, highlighting the importance of context-dependent trait expression. These traits include stomatal density (SD, 81.05%), which plays a role in regulating gas exchange, and structural support traits such as endodermis thickness (EnT, 71.70%), bundle width (BW, 46.39%), and sclerenchyma thickness (ST, 51.99%). The strong G×E contributions to these traits suggest that different genotypes respond variably to environmental variation, a hallmark of phenotypic plasticity.

**Table 2:** The contribution of genetics, environment, and their interactions to the studied traits and their ecophysiological functions. The full type-III ANOVA results for the linear Mixed-Effects models across all traits are presented in supplementary table S8

Trait	Code	G	E	G×E	Ecophysiological function
Bundle Width	BW	24.46%	29.14%	**46.39%**	Main channel for water and nutrient transport: Wider conduction bundles indicate stronger water-conducting capacity and may also support greater evapotranspiration rates and photosynthesis; positively correlated with drought resistance, especially in dry sandy environments, where thicker conduction bundles help to transport water quickly and avoid conduction blockage (embolism).
Endodermis Thickness	EnT	15.12%	13.18%	**71.70%**	Water regulation and stress response barrier: The inner surface layer may function as the endodermis, affecting the selective transport of water and solutes. Different genotypes show great differences in their responses to water stress in different environments.
Needle Length	NL	10.32%	**54.31%**	35.37%	Photosynthetic capacity and surface area adjustment: Leaf length is closely related to ambient light and water availability and is a highly plastic trait.
Needle Thickness	NT	2.98%	**70.23%**	26.79%	Water retention and light regulation: Leaf thickness will change in dry or high light conditions, which is a kind of rapid plastic response.
Phloem Thickness	PT	15.39%	**45.20%**	39.41%	Nutrient transport and growth regulation: The phloem is responsible for the transport of carbohydrates, and its thickness reflects changes in growth requirements under environmental conditions.
Stomatal Density	SD	15.42%	3.53%	**81.05%**	Photosynthesis and evapotranspiration control: High stomatal density can improve gas exchange efficiency, but it also increases water loss. It has a strong ability to adapt to different moisture environments and is highly sensitive to environmental responses. This trait also appears in the G×E category, but the majority of its variation comes from genes, indicating that this is an important adaptive trait under stable genetic control.
Stomatal Width	SW	**46.38%**	9.75%	**43.87%**	Stomatal regulation and evapotranspiration efficiency: Stomatal width affects evapotranspiration rate and gas exchange, and different genotypes may have different regulation strategies under dry and wet conditions.
Sclerenchyma Thickness	ST	**42.61%**	5.40%	**51.99%**	Mechanical support and drought adaptation: Sclerenchyma cells enhance mechanical support and also improve the stability of cell structure to resist drought or wind damage, and have structural and functional plasticity. This trait showed a strong genetic background but also appeared in the G×E category, representing a potential candidate gene for local adaptation and a manifestation of phenotypic plasticity.
Xylem Thickness	XT	**53.16%**	22.87%	23.97%	Water transport and pressure resistance: The thicker the xylem is, the more developed the water-conducting tissue is, which can improve the ability to transport water and maintain structure in arid environments.

Bold fonts indicate the main contributing factors that explain the variance of each trait

### Environmental niche comparison between ecotypes

**Fig. 5:**
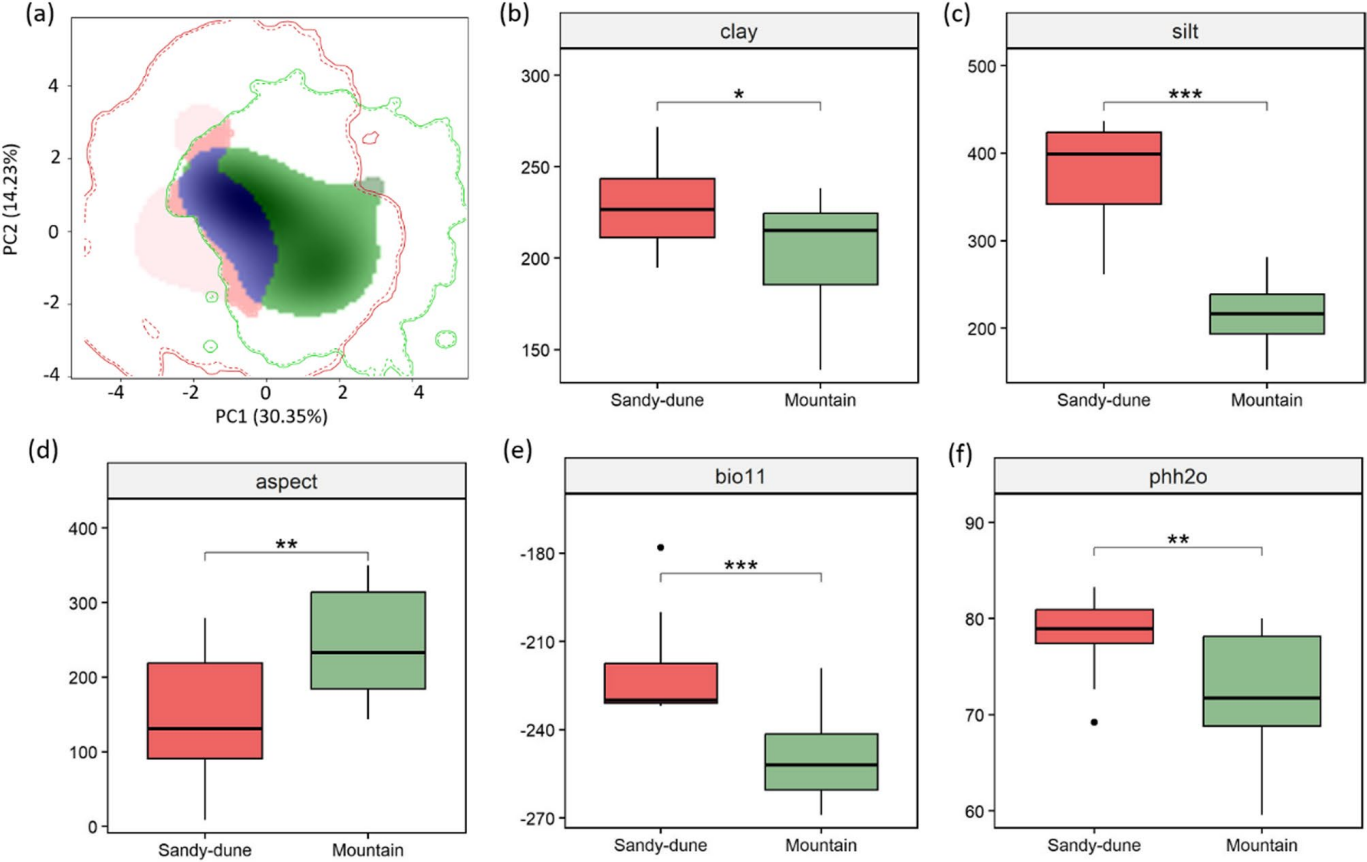
Environmental niche overlap analysis. **a** The environmental niche PCA reveals that the mountain and sandy-dune ecotypes are not completely divergent in the ecospat analysis, as shown by both the niche equivalency and niche similarity tests (Table S6). The green region represents the niche of the mountain ecotype, while the red region represents that of the sandy-dune ecotype. These two ecotypes exhibit partial divergence along PC1. **b**-**f** The *t*-tests on individual environmental variables, which reveal significant differences in (**b**) clay, (**c**) silt, (**d**) aspect, (**e**) bio11 (Mean Temperature of Coldest Quarter), and (**f**) pHH_2_O. Asterisks indicate significance levels: *p* < 0.05 (*), *p <* 0.01 (**), and *p <* 0.001 (***)

In niche comparison, we observed some overlap between the niches of different ecotypes. The calculated Schoener’s *D* value is 0.297, and Hellinger’s *I* value is 0.469, although neither result is statistically significant. In the niche equivalency test, the *p*-values for the two indices are 1 and 0.986. In the niche similarity test, the *p*-values for the two indices are 0.974 and 0.939 (Table S9). A visualized PCA plot of the niches (Fig. 5a) reveals that, although there is some overlap, the niches still differ, particularly in PC1. Factors such as aspect, bio11 (Mean Temperature of Coldest Quarter), clay, pHH_2_O, and silt appear to have a significant difference between ecotypes (Fig. 5b ~ Fig. 5f).

## Discussion

### Limited neutral differentiation but strong adaptive divergence

Frequent gene flow in Mongolian Scots pine leads to little neutral genetic differentiation between ecotypes, but 3.487% of adaptive SNPs (8,379 out of 240,288) reveal ecotype divergence linked to local climate effects on phenotypes and physiological responses. Gene flow is vital for reducing population genetic differentiation by facilitating the exchange of genetic material. It introduces new alleles, increasing genetic variation within populations and decreasing differences between them [52]. This influx helps counteract the effects of genetic drift and selection, which typically increase differentiation. Environmental pressures may restrict the gene flow of certain genes between populations, even when such flow occurs frequently (e.g., Table S7), indicating that the gene flow of these selected genes typically happens within the same cline. As an example of *Mimulus laciniatus*, gene flow is more strongly influenced by elevation than by distance, indicating isolation-by-environment (IBE), which aids populations in adapting to varying climatic conditions [53].

This study found that neutral makers showed no significant population structure between mountain and sandy-dune ecotypes.The low niche overlap between mountain and sandy-dune ecosystems (Schoener’s *D* = 0.297; Warren’s *I* = 0.469) indicates environmental differentiation, despite not statistically significant. This divergence likely restricts gene flow, allowing for the exchange of neutral alleles while limiting the movement of adaptive variants. Only about 3.5% of adaptive SNPs exhibited strong differentiation, linked to climatic and soil gradients. Typically, neutral differentiation around a single site is small, and large differentiation zones form only with low population sizes and migration rates; as multiple sites differentiate together, the differentiation tends to spread across the genome, complicating the maintenance of distinct “genomic islands” [54]. Thus, if many small-effect sites were involved in adaptation, we would expect more extensive genomic differentiation rather than just 3% of divergence sites. Fuhrmann et al. [55] showed that continuous gene flow can lead to the rapid formation of new ecotypes through allele recombination. Our findings revealed significant differentiation at only a few sites, suggesting that adaptation may depend more on simpler gene architectures or large-effect mutations.

Although we cannot directly compare the selection coefficient (*s*) and the migration rate (*m*) due to their distinct scales, the relative abundance of SNPs under divergent versus homogenizing effects provides a practical proxy. Among all genome-wide SNPs, ~ 3.5% showed high *F*_ST_ and strong associations with local environments, suggesting selection-driven divergence. In contrast, a substantial proportion of SNPs exhibited near-zero *F*_ST_, with symmetrical allele frequencies and high heterozygosity, indicative of extensive gene flow or balancing selection [56, 57]. This pattern reflects a genomic landscape where only a small subset of loci resists introgression, while the majority of the genome remains permeable, consistent with early-stage ecological divergence under gene flow [58, 59].

The notable divergence in allele frequencies observed at certain large-effect SNPs (e.g., the CEC alleles G/T) likely stems from divergent selection that maintains locally advantageous alleles in differing environmental contexts, specifically cooler, wetter montane habitats versus warmer, drier sandy dunes, despite ongoing gene flow. This pattern is consistent with the theoretical framework of selection-migration equilibrium, where the fate of adaptive alleles hinges on the balance between selection strength (*s*) and migration rate (*m*) [60, 61]. When *s* >*m*, adaptive alleles can withstand dilution and persist in local populations; conversely, when *m* >*s*, gene flow leads to the homogenization of populations. Additionally, the effects of dominance can influence this balance: dominant adaptive alleles tend to establish more easily because their fitness benefits are expressed in heterozygotes, whereas recessive adaptive alleles are often concealed and susceptible to loss during migration. This is an occurrence outlined by Haldane’s sieve and further extended to contexts with gene flow [60]. The observed biases in allele frequencies may thus reflect asymmetric constraints on adaptive gene flow, where specific variants are maintained locally while others are hindered from disseminating across different ecotypes. Such loci may also impact G×E interactions and phenotypic plasticity, playing crucial roles in the survival of ecotypes amidst environmental heterogeneity and ongoing migration.

### Genomic architecture of adaptation: polygenic shifts vs. genomic Islands

Polygenic local adaptation can promote speciation when adaptive loci are linked through linkage disequilibrium (LD) or specific genomic structures, allowing for synergistic selection on multiple traits that reinforce ecological isolation [23, 24]. If adaptation relies on a few large-effect loci, known as genomic islands, these can significantly contribute to ecotype divergence, particularly under spatially varying selection [29].

Although many environment-associated SNPs have high LFMM |*z*|-scores (>3), their broad genomic distribution suggests a predominantly polygenic architecture of adaptation, with only a small number of candidate large-effect loci such as the CEC gene, potentially contributing to more localized genomic differentiation. Polygenic local adaptation can drive ecological divergence, especially when adaptive loci are hypothesized to be clustered or located in regions of reduced recombination, which helps maintain locally adapted haplotypes despite gene flow [29, 62]. Such clustering has been observed in other plant systens and may arise through mechanisms such as chromosomal inversions or linkage disequilibrium [63], although we currently lack direct evidence of these processes in our study system. Regions of low recombination may show elevated divergence due to both direct and linked selection [64]. While our data fo not allow us to confirm or reject the presence of genomic islands, the strong association of high-effect SNPs with environmental gradients is consistent with divergent selection acting on a subset of loci.

We identified a key locus associated with a gene related to cation exchange capacity (CEC), crucial for soil nutrient retention. The G allele frequency was 0.667% in the mountain ecotype, while the T allele frequency was 0.792% in the sandy-dune ecotype. These differences suggest adaptations to soil fertility challenges [65]. Sandy soils have low CEC, which limits nutrient retention and increases the risk of leaching essential cations like K⁺, Ca²⁺, and Mg²⁺ [65, 66]. Plants in these environments may need better ion transport efficiency and root-mediated pH buffering [67]. The associated allele may enhance performance in nutrient-poor conditions by improving cation uptake or minimizing nutrient loss, indicating local adaptations to specific soil chemistry and underscoring the role of soil factors in driving plant ecotype divergence [68]. This may serve as an example of ecological genomics, connecting genomic variation to adaptive strategies in diverse soil conditions.

Large-effect loci can also serve as “genomic islands of divergence” [69, 70], acting as focal points for adaptation. Although this study does not provide direct evidence of clustering or reduced recombination, the high-effect SNPs observed, such as the CEC-associated locus, suggest strong selection related to soil nutrient retention in sandy habitats. Such loci are crucial for maintaining adaptive divergence under high gene flow, where only strong-effect alleles can resist dilution [60, 61]. In contrast, polygenic adaptive divergence may be particularly vulnerable to swamping by gene flow unless reinforced by genotype–environment covariance or clustering of adaptive loci, which can enhance their collective resistance to recombination and migration [71, 72].

Our findings on high-impact adaptive SNPs indicate that even in conifers with significant gene flow, genomic organization can facilitate ecological divergence and potentially lead to speciation. Butlin and Faria [73] suggest that local adaptation may contribute to speciation through reproductive isolation. For instance, differences in adaptation between mountain and sandy-dune habitats could create partial ecological isolation, signaling early stages of speciation. While initial outcrossing between ecotypes may enhance survival by increasing genetic diversity, maladaptive alleles could lead to reproductive barriers in their offspring [74]. Theoretical models show that polygenic adaptation faces challenges under gene flow. In high-migration landscapes, small-effect alleles can be overwhelmed unless adaptive loci are tightly linked. Genotype–environment covariance can help maintain adaptive combinations. Thus, the interplay of gene flow, selection, and genomic architecture likely determines whether polygenic divergence contributes to speciation [75, 76]. Our findings highlight how large-effect loci and a broader polygenic background can work together to promote ecotype divergence in diverse environments.

### Phenotypic plasticity and genotype-by-environment interactions

We identified several sets of large-effect loci with environmental adaptation [24, 29]. Based on GEA analyses, we identified a subset (~ 3%) of SNPs with extreme environmental associations, representing the upper tail of the genome-wide effect-size distribution. The effect-size spectrum showed a strongly peaked shape, indicating that a limited number of loci contribute disproportionately to environment-associated genomic divergence. These results provide empirical evidence for the presence of large-effect loci associated with environmental gradients. Such loci may confer to ecotype-specific fitness advantages and could reduce effective recombination among co-adapted alleles. However, the observed morphological variation also reveals significant contributions from environmental effects and G×E interactions, suggesting a substantial role for phenotypic plasticity alongside genetic differentiation. In traits such as endodermis thickness, stomatal width and density, and sclerenchyma tissue thickness, G×E effects were particularly pronounced, indicating that different genotypes exhibit distinct phenotypic responses across environments. This aligns with recent studies showing that G×E effects often map to polygenic axes of variation and can underpin environment-specific trait expression [22, 55, 77]. Therefore, our results suggest that ecotype divergence is shaped by a combination of genic adaptation and plastic responses, rather than a single dominant mode of differentiation.

Our results revealed that genetically based traits are mostly related to water transport and mechanical support, meaning that these traits are important targets for selection and adaptation [78, 79]. In contrast, gas exchange, water regulation, mechanical support, and other related traits are mainly affected by G×E interaction, highlighting the role of phenotypic plasticity. This suggests that these traits exhibit different responses under varying habitat conditions (e.g., mountainous vs. sandy-dune habitats), which are potential indicators of local adaptation. However, the characteristics of needle morphology and nutrient transport are more easily adjusted to environmental changes, exhibiting a broader range of reaction norms. It is particularly noteworthy that water transport regulation and mechanical support are significantly influenced by genetic factors (G) and gene-environment interactions (G×E). This suggests that, beyond the selection of adaptive differences in various habitats, these factors also demonstrate a flexible strategy for adjustment in response to environmental changes. Recent theory suggests that G×E loci may provide a reservoir of adaptive potential under changing environments, as their environment-specific effects can facilitate rapid shifts in trait expression before genetic assimilation occurs [22, 80]. This flexibility highlights their capacity for plasticity in adapting to diverse environments.

This suggests that plastic or G×E-modulated traits may represent transient stages in the trajectory toward fixed divergence. Under continued divergent selection, genotype-specific reaction norms could eventually canalize into genetic differentiation, i.e., the plasticity-first hypothesis [81]. This process may allow populations to initially cope with novel habitats via plasticity, while gradually fixing adaptive trait values through selection on standing variation or new mutations. The coexistence of genetically determined, environmentally induced, and G×E-mediated traits in populations raises questions about functional or developmental constraints. Some traits may be positioned along a genetic plasticity continuum [82], where selection targets both the trait values and their environmental sensitivity. However, not all plastic traits necessarily canalize into fixed genetic differences. The maintenance of plasticity may reflect balancing selection favoring flexibility in heterogeneous environments or the costs of maintaining plasticity [83].

Traits related to water transport and tissue integrity, such as xylem thickness and sclerenchyma, show stronger genetic control, whereas leaf size and phloem traits are mainly influenced by environmental factors. This suggests that adaptive divergence in Mongolian Scots pine is trait-specific. Hydraulic and structural traits form resilient genetic modules that maintain tree function under stress [84, 85], while photosynthetic and transport traits remain flexible for environmental adaptation [86]. Such modularity likely reflects varying selection pressures in different habitats. We thus infer that adaptive divergence appears trait-specific, shaped by modularity and varying resistance to environmental change. Notably, in the sandy-dune ecotype, traits promote increased secondary growth and reduced water loss, emphasizing defense. In contrast, the mountain ecotype focuses on rapid primary growth and minimizes secondary growth. This represents a growth-defense trade-off: mountain ecotypes invest in flexible traits for favorable conditions, while sandy-dune ecotypes develop fixed adaptations in harsh environments. Thus, these traits help balance growth and drought resistance, becoming important targets for selection in diverse landscapes.

### Conservation and management implications under climate change

Traditionally, forest breeding and provenance zones have relied on field trials and phenotypic adaptability, emphasizing the concept of "local provenance." However, Yu et al. [87] proposed landscape genomics as a framework for delineating breeding zones based on adaptive genetic variation linked to environmental factors, such as climate. Since neutral markers may underestimate ecotype differentiation, conservation strategies should prioritize adaptive loci and ecological environments. For Mongolian Scots pine, this means defining provenance zones aligned with distinct habitats, such as mountainous vs. sandy-dune areas, or the presence of key adaptive genes, ensuring that transplanted seedlings are matched to future climatic conditions [88]. While initial hybridization between ecotypes may provide short-term benefits, evidence from Torrey pine suggests that maladaptive gene flow can reduce fitness in later generations [74]. This underscores the need to protect the unique adaptive diversity of each ecotype by maintaining humid high-altitude habitats for mountain populations and stable arid environments for sandy-dune populations. Looking forward, integrating genomic insights with climate change projections could enable dynamic conservation planning to balance local adaptation and genetic diversity in forest trees [87–89].

## Supplementary Information

Supplementary Material 1

## Data Availability

The SLAF-seq data generated in this study have been deposited into the European Nucleotide Archive (ENA) and can be found at the project accession PRJEB94076 with the sample accession no. ERS25272215~ERS25272314. Alldatasets used and/or analysed during the current study are available from the corresponding authors on reasonable request.
