# Supplementary material for: Ecotypic divergence in Mongolian Scots pine persists via large-effect genetic adaptation and phenotypic plasticity
Source: BMC Plant Biol. 2025 Nov 26;25:1642. doi: 10.1186/s12870-025-07695-0 (PMC12659373; doi:10.1186/s12870-025-07695-0)
Supplement: Supplementary file 1 — Supplementary Material 1 [file 12870_2025_7695_MOESM1_ESM.docx]

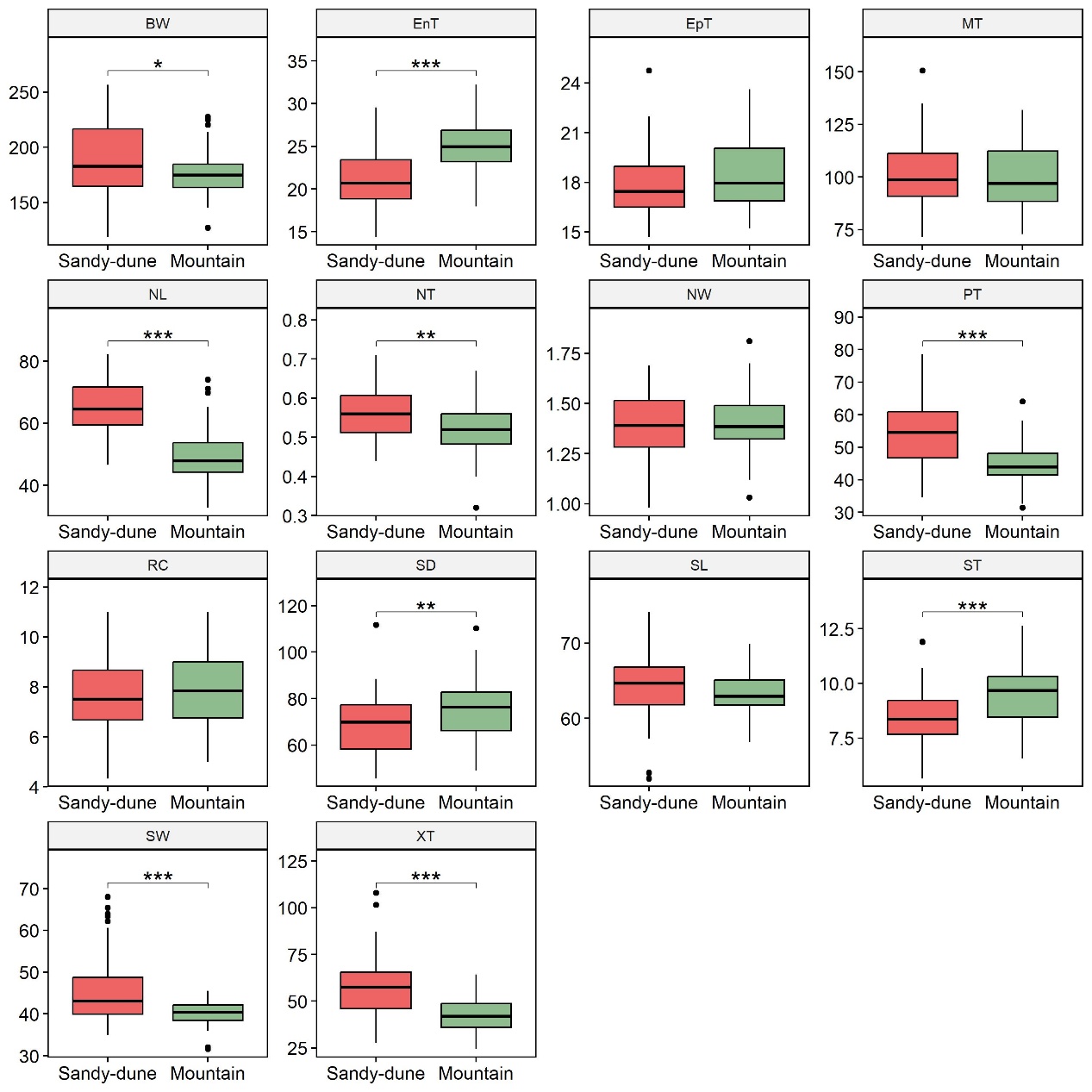


**Fig. S1** Results of two-sample *t*-tests comparing phenotypic traits between sand-dune and mountain ecotypes. Traits include conduction bundle width (BW), endodermis thickness (EnT), epidermis thickness (EpT), mesophyll thickness (MT), needle length (NL), needle thickness (NT), needle width (NW), phloem thickness (PT), resin canal number (RC), stomatal density (SD), stomatal length (SL), sclerenchyma thickness (ST), stomatal width (SW), and xylem thickness (XT). Nine traits showed significant differences between ecotypes: BW (*p* < 0.05); NT and SD (*p* < 0.01); and EnT, NL, PT, SL, SW and XT (*p* < 0.001). Asterisks indicate significance levels: *p* < 0.05 (*)*, p* < 0.01 (**), and *p* < 0.001 (***).


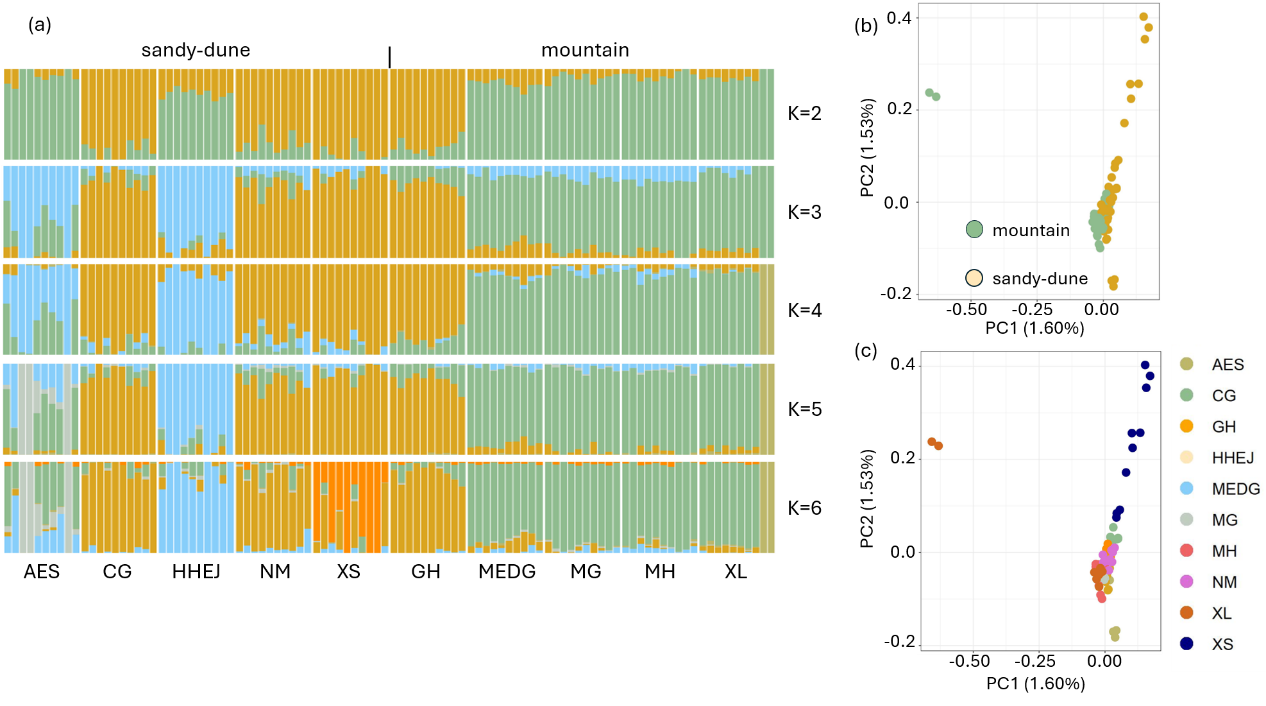


**Fig. S2** Genetic structure based on the neutral SNP dataset. (a) In the sNMF analysis, AES and HHEJ shared a similar genetic component with the mountain ecotype, while GH shared the component with the sandy-dune ecotype when *K* = 2. AES and HHEJ formed a unique cluster when *K* > 3, and AES showed signs of admixture when *K* > 5. XS formed a distinct component at *K* = 6, and when *K* > 4, two individuals from XL exhibited a unique genetic component. (b) In the PCA labeled by ecotype, the two ecotypes clustered into a single group, showing no clear separation. (c) In the PCA labeled by population, two individuals from XL were separated from the others along PC1, while XS samples diffused from other populations along PC2


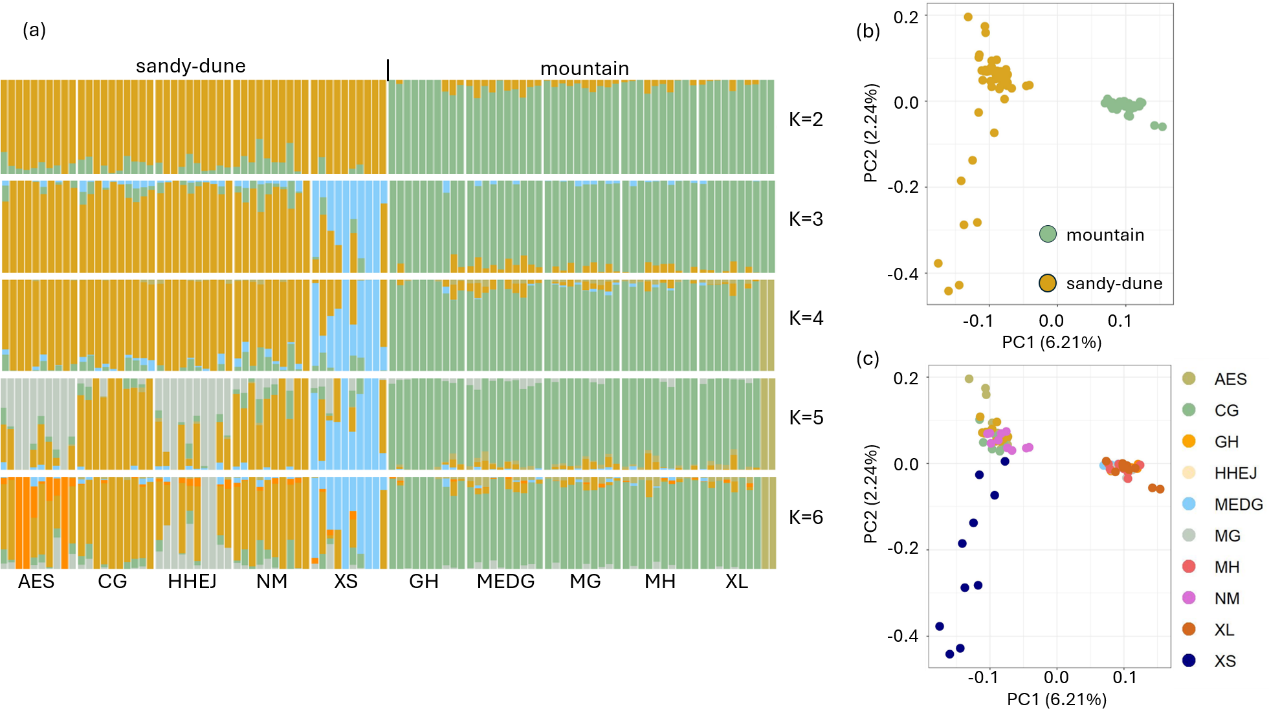


**Fig. S3** Genetic structure based on *F*_ST_-outlier SNPs dataset.

(a) In the sNMF analysis, a clear divergence between ecotypes was observed at *K* = 2. XS formed a distinct genetic component when *K* > 3. AES and HHEJ formed a unique genetic cluster at *K* = 5, and AES exhibited another distinct genetic component at *K* = 6. Similar to the pattern in the neutral SNP dataset, two individuals from XL displayed a different genetic component when *K* > 4. (b) In the PCA labeled by ecotype, the two ecotypes were clearly separated along PC1, indicating strong genetic differentiation in the outlier SNPs. (c) In the PCA labeled by population, two individuals from XL were separated from the mountain ecotype group along PC1, while XS showed a dispersed pattern along PC2.


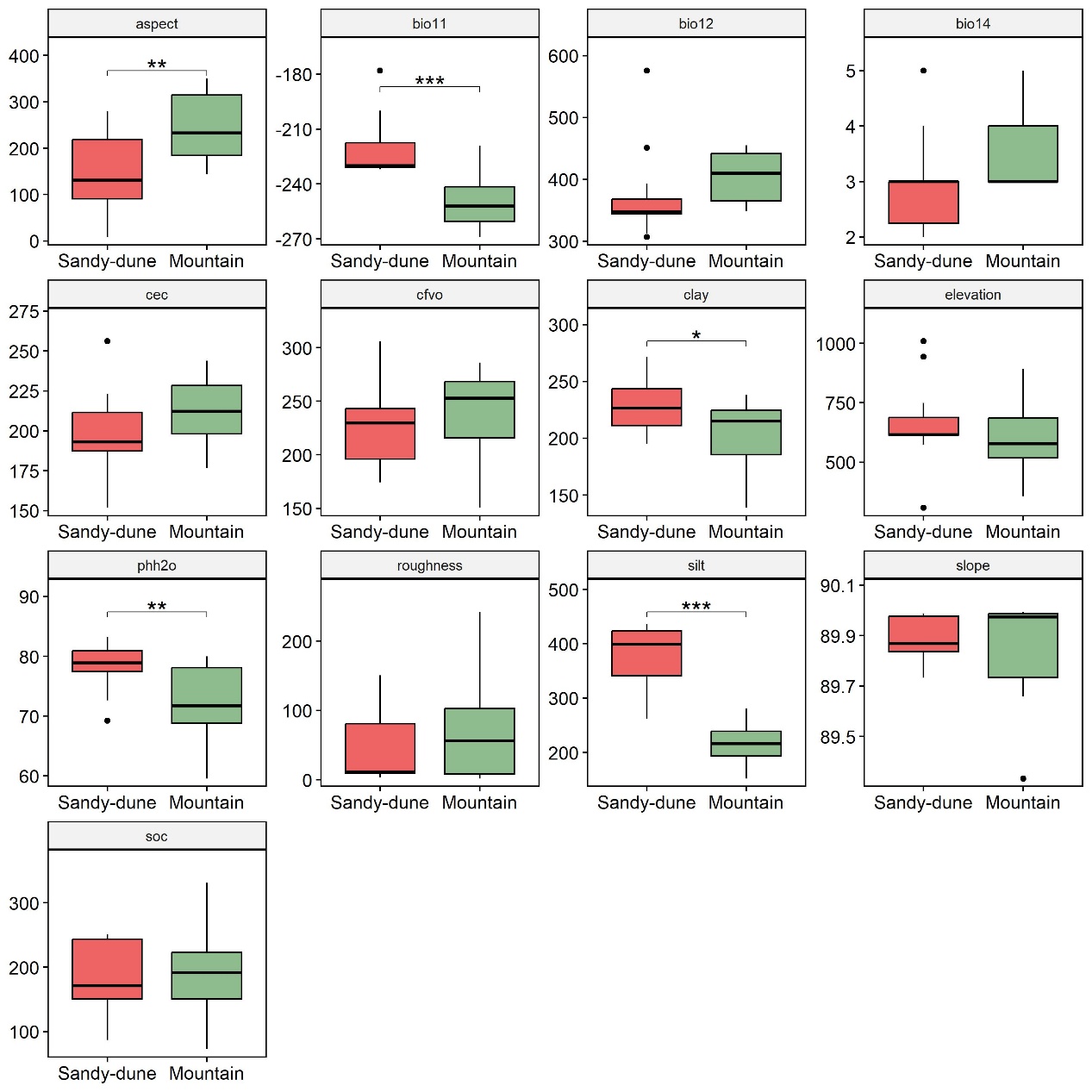


**Fig. S4** Comparison of environmental variables between mountain and sandy-dune ecotypes based on two-sample *t*-tests. Variables include bioclimatic factors such as bio11 (mean temperature of coldest quarter), bio12 (annual precipitation), and bio14 (precipitation of driest month); edaphic attributes such as cec (cation exchange capacity), cfvo (volumetric fraction of coarse fragments), pHH_2_O (soil pH), soc (soil organic carbon content), proportion of clay (< 0.002 mm) and silt (0.002–0.05 mm); and topographic metrics including aspect, elevation, roughness, slope. All environmental variables included in the analysis had VIF values < 10 to avoid multicollinearity. Five environmental variables showed significant differences between ecotypes: clay (*p* < 0.05); aspect and phh2o (*p* < 0.01); and bio11 and slit (*p* < 0.001). Asterisks indicate significance levels: *p* < 0.05 (*)*, p*< 0.01 (**), and *p*< 0.001 (***).


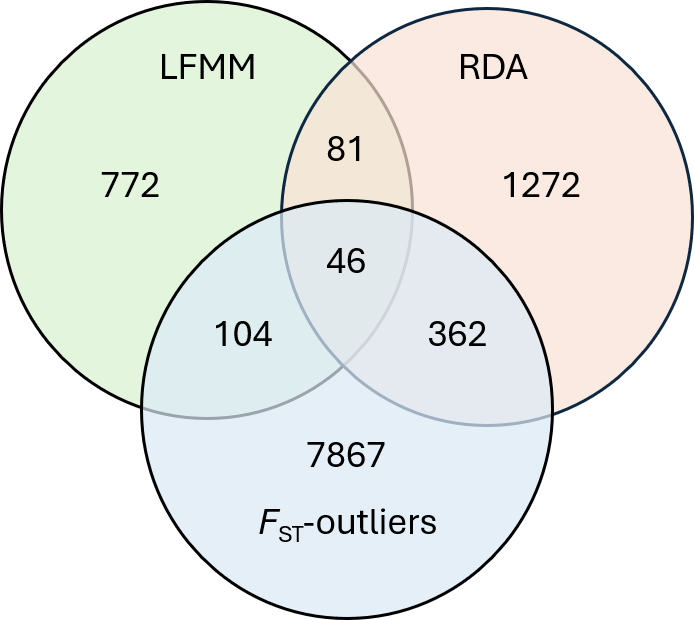


**Fig. S5** Venn diagram of environment-associated SNPs and *F*_ST_-outlier SNPs. The LFMM analysis identified 1,003 environment-associated SNPs, while RDA detected 1,761. The intersection of these two GEA approaches yielded 127 SNPs, which are considered robust environment-associated candidates. Among these, 46 SNPs were also identified as *F*_ST_-outliers, potentially involved in local adaptation between ecotypes.

**Table S1** Summary of functional and adaptive significance of nine selected traits for Gene × Environment interaction mixed models analysis

| Category | Trait(s) | Physiological and ecological significance |
| --- | --- | --- |
| Stomatal traits | SD (Stomatal Density), SW (Stomatal Width) | Control gas exchange and transpiration efficiency; reflect plant responses to water availability and atmospheric conditions. |
| Leaf structural traits | NL (Needle Length), NT (Needle Thickness) | Influence leaf surface area, photosynthetic potential, and water retention; highly plastic traits sensitive to environmental stress. |
| Conductive tissue traits | XT (Xylem Thickness), PT (Phloem Thickness), BW (Bundle Width) | Determine hydraulic and assimilate transport efficiency; critical for drought tolerance and maintaining physiological performance. |
| Supportive tissue | ST (Sclerenchyma Thickness) | Enhance mechanical stability under harsh conditions (e.g., wind, heat); prevent vessel collapse and support long-term function. |
| Barrier tissue | EnT (Endodermis Thickness) | Acts as a selective barrier to water and solute movement; potentially involved in drought resistance and internal water regulation. |

**Table S2** Environmental variables retained after stepwise multicollinearity filtering using vifstep() (VIF threshold = 10) in R package usdm. The table shows the final subset of variables and their variance inflation factor (VIF) values.

| **Category** | **Variables** | **VIF** |
| --- | --- | --- |
| Bioclimatic variables | bio11 | 1.077 |
|  | bio12 | 1.104 |
|  | bio14 | 1.054 |
| Edaphic attributes | cec | 1.529 |
|  | cfvo | 4.091 |
|  | clay | 1.681 |
|  | phh2o | 4.296 |
|  | silt | 1.447 |
|  | soc | 1.756 |
| Topographic metrics | aspect | 1.528 |
|  | elevation | 1.465 |
|  | roughness | 3.786 |
|  | slope | 4.246 |

**Table S3** Results of principal component analysis (PCA) on environmental variables.

The first two principal components (Env_PC1 and Env_PC2), which together explained the largest proportion of total variance, were selected as environmental covariates (E effects) in the subsequent modelling.

|  | **Env_PC1** | **Env_PC2** | **Env_PC3** | **Env_PC4** | **Env_PC5** | **Env_PC6** | **Env_PC7** | **Env_PC8** | **Env_PC9** | **Env_PC10** |
| --- | --- | --- | --- | --- | --- | --- | --- | --- | --- | --- |
| Standard deviation | 2.12 | 1.73 | 1.36 | 1.19 | 0.90 | 0.81 | 0.70 | 0.46 | 0.28 | 0.00 |
| Proportion of Variance | 0.35 | 0.23 | 0.14 | 0.11 | 0.06 | 0.05 | 0.04 | 0.02 | 0.01 | 0.00 |
| Cumulative Proportion | 0.35 | 0.58 | 0.72 | 0.83 | 0.89 | 0.94 | 0.98 | 0.99 | 1.00 | 1.00 |

**Table S4** Results of MANOVA assessing environmental differentiation between mountain and sandy-dune ecotypes based on the first three principal components (PC1–PC3), derived from a PCA of 13 environmental variables with variance inflation factor (VIF) ≤ 10.

| **Term** | **df** | **Pillai's trace** | ***F*** | **num Df** | **den df** | ***p*** |
| --- | --- | --- | --- | --- | --- | --- |
| Ecotype | 1 | 0.676 | 4.179 | 3 | 6 | 0.065 |
| Residuals | 8 |  |  |  |  |  |

**Table S5** Results of PERMANOVA comparing environmental differences between mountain and sandy-dune ecotypes based on the first three PCA axes, using 999 permutations.

| **Term** | **df** | **Sum of sq** | **R2** | ***F*** | ***p*** |
| --- | --- | --- | --- | --- | --- |
| Model | 1 | 17.985 | 0.214 | 2.179 | 0.068 |
| Residual | 8 | 66.020 | 0.786 |  |  |
| Total | 9 | 84.004 | 1 |  |  |

**Table S6** Result of intersection of *F*_ST_-outliers and environment-associated SNPs. The last two columns are environment predictors of two GEA approaches.

| **Chromosome** | **Position** | **Ref_Allele** | **Alt_Allele** | **Env_LFMM** | **Env_RDA** |
| --- | --- | --- | --- | --- | --- |
| Marker67983 | 102 | T | G | slope | roughness |
| Marker69063 | 5 | G | C | slope | roughness |
| Marker70022 | 188 | C | A | slope | roughness |
| Marker75551 | 181 | G | T | slope | roughness |
| Marker77933 | 250 | G | A | slope | roughness |
| Marker80741 | 227 | A | T | silt | clay |
| Marker82950 | 143 | A | G | slope | roughness |
| Marker86237 | 89 | T | C | slope | roughness |
| Marker87164 | 66 | A | G | slope | roughness |
| Marker88803 | 89 | G | C | cec | roughness |
| Marker90812 | 93 | T | C | slope | roughness |
| Marker99775 | 199 | T | G | slope | roughness |
| Marker107129 | 230 | C | A | bio12 | roughness |
| Marker111593 | 182 | G | A | slope | roughness |
| Marker113273 | 236 | G | T | slope | roughness |
| Marker114998 | 252 | T | C | slope | roughness |
| Marker116298 | 101 | A | G | bio12 | roughness |
| Marker117584 | 124 | G | T | cec | cec |
| Marker121653 | 36 | A | C | bio11 | clay |
| Marker124377 | 119 | A | C | slope | roughness |
| Marker126017 | 12 | A | G | slope | roughness |
| Marker135167 | 102 | A | G | slope | roughness |
| Marker205346 | 61 | C | A | slope | roughness |
| Marker231646 | 182 | A | G | slope | roughness |
| Marker258859 | 94 | T | A | slope | roughness |
| Marker281175 | 157 | G | C | silt | clay |
| Marker303669 | 105 | G | A | slope | roughness |
| Marker330530 | 239 | G | A | slope | roughness |
| Marker388283 | 40 | T | C | slope | roughness |
| Marker451052 | 27 | A | G | slope | roughness |
| Marker553439 | 211 | T | G | slope | roughness |
| Marker557454 | 93 | T | G | clay | bio12 |
| Marker578792 | 166 | C | G | slope | roughness |
| Marker587023 | 9 | A | G | slope | roughness |
| Marker889844 | 34 | A | G | slope | roughness |
| Marker942492 | 201 | A | T | silt | clay |
| Marker964985 | 58 | T | G | slope | roughness |
| Marker1170596 | 209 | T | G | slope | roughness |
| Marker1381979 | 232 | G | A | bio12 | roughness |
| Marker1766991 | 94 | A | C | slope | roughness |
| Marker2063531 | 80 | T | C | silt | clay |
| Marker2861842 | 113 | C | G | slope | roughness |
| Marker6182908 | 237 | A | G | slope | roughness |
| Marker12327207 | 203 | T | C | bio14 | roughness |
| Marker14623750 | 157 | A | G | slope | roughness |
| Marker14623750 | 159 | T | C | slope | roughness |

**Table S7**

Results of BLAST analysis of environment-associated sequences against the NCBI database. We performed BLAST searches to identify the genes associated with the environment-associated sequences, and inferred their functions based on annotations and information from published studies.

| **Chromosome** | **E-value** | **Gene** | **Function** | **References** |
| --- | --- | --- | --- | --- |
| Marker70022 | 1.83E-33 | *KOR1* | Endosperm cellularization,  Cell elongation, Cell wall formation,  Downregulation causing dwarf phenotype,  Delayed germination. | (Zuo *et al.*, 2000; Nairn *et al.*, 2008; Shang *et al.*, 2015) |
| Marker181747 | 2.34E-42 | *KOR1* |  |  |
| Marker73476 | 3.11E-21 | *SuSy1* | Sucrose translocation in phloem,  Sucrose accumulation, Promote plant growth | (Nairn *et al.*, 2008; Julius *et al.*, 2017; Durand *et al.*, 2018; Noman *et al.*, 2022) |
| Marker77281 | 3.09E-26 | *SuSy1* |  |  |
| Marker73476 | 1.12E-20 | *Lac8* | Xylem development, especial in root in *Arabidopsis* | (Palle *et al.*, 2011; Berthet *et al.*, 2012) |
| Marker388283 | 1.44E-24 | *MYB8* | Lignification, Promote cell wall secondary growth in *Pinus taeda* | (Bomal *et al.*, 2008; Palle *et al.*, 2011; Ding *et al.*, 2024) |
| Marker557454 | 6.83E-08 | *CesA1* | Accumulate primary wall cellulose | (Burn *et al.*, 2002; Nairn *et al.*, 2008; Palle *et al.*, 2011; Li *et al.*, 2024) |
| Marker77281 | 6.64E-28 | *CCoAOMT* | Evolved in lignin monomer synthesis | (Li *et al.*, 2024) |

**Table S8** Full Type III ANOVA results for Linear Mixed-Effects Models across all traits.

| **Trait** | **Effect** | **Sum Sq** | **Mean Sq** | **NumDF** | **DenDF** | ***F*** | ***p*** | **PctExp** |
| --- | --- | --- | --- | --- | --- | --- | --- | --- |
| BW | PC1 | 854.05 | 854.05 | 1 | 88 | 1.0673 | 0.3044 | 16.85% |
| BW | PC2 | 253.32 | 253.32 | 1 | 88 | 0.3166 | 0.5751 | 5.00% |
| BW | PC3 | 132.22 | 132.22 | 1 | 88 | 0.1652 | 0.6854 | 2.61% |
| BW | Env_PC1 | 250.01 | 250.01 | 1 | 88 | 0.3124 | 0.5776 | 4.93% |
| BW | Env_PC2 | 1226.76 | 1226.76 | 1 | 88 | 1.5331 | 0.2189 | 24.21% |
| BW | PC1: Env_PC1 | 804.02 | 804.02 | 1 | 88 | 1.0048 | 0.3189 | 15.87% |
| BW | PC1: Env_PC2 | 0.43 | 0.43 | 1 | 88 | 0.0005 | 0.9816 | 0.01% |
| BW | PC2: Env_PC1 | 500.56 | 500.56 | 1 | 88 | 0.6256 | 0.4311 | 9.88% |
| BW | PC2: Env_PC2 | 324.36 | 324.36 | 1 | 88 | 0.4054 | 0.526 | 6.40% |
| BW | PC3: Env_PC1 | 5.63 | 5.63 | 1 | 88 | 0.007 | 0.9333 | 0.11% |
| BW | PC3: Env_PC2 | 715.97 | 715.97 | 1 | 88 | 0.8947 | 0.3468 | 14.13% |
| EnT | PC1 | 0.0058 | 0.0058 | 1 | 62.086 | 0.0007 | 0.9792 | 0.02% |
| EnT | PC2 | 2.92 | 2.92 | 1 | 77.015 | 0.3434 | 0.5596 | 12.46% |
| EnT | PC3 | 0.6171 | 0.6171 | 1 | 40.745 | 0.0726 | 0.789 | 2.63% |
| EnT | Env_PC1 | 0.5449 | 0.5449 | 1 | 9.449 | 0.0641 | 0.8056 | 2.33% |
| EnT | Env_PC2 | 2.542 | 2.542 | 1 | 16.931 | 0.299 | 0.5917 | 10.85% |
| EnT | PC1: Env_PC1 | 0.0009 | 0.0009 | 1 | 78.983 | 0.0001 | 0.9916 | 0.00% |
| EnT | PC1: Env_PC2 | 0.0047 | 0.0047 | 1 | 80.177 | 0.0006 | 0.9813 | 0.02% |
| EnT | PC2: Env_PC1 | 7.5375 | 7.5375 | 1 | 82.303 | 0.8865 | 0.3492 | 32.18% |
| EnT | PC2: Env_PC2 | 0.0262 | 0.0262 | 1 | 88 | 0.0031 | 0.9558 | 0.11% |
| EnT | PC3: Env_PC1 | 1.6176 | 1.6176 | 1 | 84.201 | 0.1903 | 0.6638 | 6.91% |
| EnT | PC3: Env_PC2 | 7.609 | 7.609 | 1 | 54.073 | 0.8949 | 0.3484 | 32.48% |
| NL | PC1 | 2.91 | 2.91 | 1 | 51.455 | 0.0451 | 0.83263 | 0.40% |
| NL | PC2 | 9.43 | 9.43 | 1 | 68.631 | 0.1463 | 0.70331 | 1.30% |
| NL | PC3 | 62.66 | 62.66 | 1 | 30.682 | 0.9719 | 0.33191 | 8.62% |
| NL | Env_PC1 | 9.61 | 9.61 | 1 | 10.892 | 0.1491 | 0.70686 | 1.32% |
| NL | Env_PC2 | 385.12 | 385.12 | 1 | 20.398 | 5.9738 | 0.02372* | 52.99% |
| NL | PC1: Env_PC1 | 14.82 | 14.82 | 1 | 74.411 | 0.2299 | 0.63302 | 2.04% |
| NL | PC1: Env_PC2 | 34.9 | 34.9 | 1 | 72.431 | 0.5413 | 0.46428 | 4.80% |
| NL | PC2: Env_PC1 | 27.74 | 27.74 | 1 | 77.875 | 0.4303 | 0.51378 | 3.82% |
| NL | PC2: Env_PC2 | 166.76 | 166.76 | 1 | 87.34 | 2.5867 | 0.11138 | 22.94% |
| NL | PC3: Env_PC1 | 3.04 | 3.04 | 1 | 79.791 | 0.0471 | 0.82868 | 0.42% |
| NL | PC3: Env_PC2 | 9.84 | 9.84 | 1 | 45.357 | 0.1527 | 0.6978 | 1.35% |
| NT | PC1 | 0.00158 | 0.00158 | 1 | 45.86 | 0.5832 | 0.44898 | 2.73% |
| NT | PC2 | 2.5E-05 | 2.5E-05 | 1 | 63.567 | 0.0092 | 0.92406 | 0.04% |
| NT | PC3 | 0.00012 | 0.00012 | 1 | 26.171 | 0.0443 | 0.83485 | 0.21% |
| NT | Env_PC1 | 0.00579 | 0.00579 | 1 | 11.463 | 2.1312 | 0.17119 | 9.97% |
| NT | Env_PC2 | 0.03495 | 0.03495 | 1 | 21.917 | 12.8729 | 0.001646** | 60.25% |
| NT | PC1: Env_PC1 | 0.00244 | 0.00244 | 1 | 71.952 | 0.8976 | 0.34659 | 4.20% |
| NT | PC1: Env_PC2 | 0.00089 | 0.00089 | 1 | 67.521 | 0.3289 | 0.56822 | 1.54% |
| NT | PC2: Env_PC1 | 0.00438 | 0.00438 | 1 | 75.222 | 1.6129 | 0.208 | 7.55% |
| NT | PC2: Env_PC2 | 0.00309 | 0.00309 | 1 | 86.447 | 1.1391 | 0.28882 | 5.33% |
| NT | PC3: Env_PC1 | 0.00044 | 0.00044 | 1 | 76.964 | 0.1606 | 0.68976 | 0.75% |
| NT | PC3: Env_PC2 | 0.0043 | 0.0043 | 1 | 41.128 | 1.5852 | 0.21511 | 7.42% |
| PT | PC1 | 12.6 | 12.6 | 1 | 66.44 | 0.1997 | 0.65641 | 1.46% |
| PT | PC2 | 57.788 | 57.788 | 1 | 80.058 | 0.9159 | 0.34143 | 6.72% |
| PT | PC3 | 62.062 | 62.062 | 1 | 45.44 | 0.9836 | 0.32655 | 7.21% |
| PT | Env_PC1 | 129.54 | 129.54 | 1 | 8.383 | 2.0531 | 0.18811 | 15.05% |
| PT | Env_PC2 | 259.375 | 259.375 | 1 | 14.679 | 4.1109 | 0.06116 | 30.14% |
| PT | PC1: Env_PC1 | 137.835 | 137.835 | 1 | 80.835 | 2.1846 | 0.14328 | 16.02% |
| PT | PC1: Env_PC2 | 73.482 | 73.482 | 1 | 82.836 | 1.1646 | 0.28364 | 8.54% |
| PT | PC2: Env_PC1 | 0.183 | 0.183 | 1 | 83.945 | 0.0029 | 0.95716 | 0.02% |
| PT | PC2: Env_PC2 | 67.774 | 67.774 | 1 | 87.873 | 1.0742 | 0.30285 | 7.88% |
| PT | PC3: Env_PC1 | 3.045 | 3.045 | 1 | 85.701 | 0.0483 | 0.82664 | 0.35% |
| PT | PC3: Env_PC2 | 56.834 | 56.834 | 1 | 57.858 | 0.9008 | 0.34652 | 6.60% |
| SD | PC1 | 13.58 | 13.58 | 1 | 88 | 0.0856 | 0.7706 | 0.72% |
| SD | PC2 | 69.31 | 69.31 | 1 | 88 | 0.4367 | 0.5104 | 3.67% |
| SD | PC3 | 207.88 | 207.88 | 1 | 88 | 1.3099 | 0.2555 | 11.02% |
| SD | Env_PC1 | 51.24 | 51.24 | 1 | 88 | 0.3229 | 0.5713 | 2.72% |
| SD | Env_PC2 | 15.38 | 15.38 | 1 | 88 | 0.0969 | 0.7563 | 0.82% |
| SD | PC1: Env_PC1 | 194.99 | 194.99 | 1 | 88 | 1.2287 | 0.2707 | 10.34% |
| SD | PC1: Env_PC2 | 432.89 | 432.89 | 1 | 88 | 2.7276 | 0.1022 | 22.95% |
| SD | PC2: Env_PC1 | 341.15 | 341.15 | 1 | 88 | 2.1496 | 0.1462 | 18.09% |
| SD | PC2: Env_PC2 | 339.83 | 339.83 | 1 | 88 | 2.1413 | 0.1469 | 18.02% |
| SD | PC3: Env_PC1 | 194.58 | 194.58 | 1 | 88 | 1.2261 | 0.2712 | 10.32% |
| SD | PC3: Env_PC2 | 25.44 | 25.44 | 1 | 88 | 0.1603 | 0.6899 | 1.35% |
| SW | PC1 | 30.224 | 30.224 | 1 | 87.305 | 2.8977 | 0.09227 | 18.51% |
| SW | PC2 | 37.885 | 37.885 | 1 | 85.89 | 3.6322 | 0.06002 | 23.20% |
| SW | PC3 | 7.632 | 7.632 | 1 | 87.999 | 0.7317 | 0.39466 | 4.67% |
| SW | Env_PC1 | 0.931 | 0.931 | 1 | 7.265 | 0.0892 | 0.7735 | 0.57% |
| SW | Env_PC2 | 14.987 | 14.987 | 1 | 8.944 | 1.4368 | 0.26145 | 9.18% |
| SW | PC1: Env_PC1 | 0.352 | 0.352 | 1 | 86.522 | 0.0337 | 0.85471 | 0.22% |
| SW | PC1: Env_PC2 | 29.844 | 29.844 | 1 | 85.245 | 2.8613 | 0.09439 | 18.28% |
| SW | PC2: Env_PC1 | 3.066 | 3.066 | 1 | 85.77 | 0.294 | 0.5891 | 1.88% |
| SW | PC2: Env_PC2 | 2.289 | 2.289 | 1 | 83.01 | 0.2194 | 0.64071 | 1.40% |
| SW | PC3: Env_PC1 | 2.814 | 2.814 | 1 | 84.859 | 0.2698 | 0.60484 | 1.72% |
| SW | PC3: Env_PC2 | 33.273 | 33.273 | 1 | 87.899 | 3.19 | 0.07754 | 20.38% |
| ST | PC1 | 5.6287 | 5.6287 | 1 | 20.345 | 4.8519 | 0.0393* | 21.33% |
| ST | PC2 | 5.6033 | 5.6033 | 1 | 35.08 | 4.8301 | 0.03466* | 21.23% |
| ST | PC3 | 0.0139 | 0.0139 | 1 | 9.903 | 0.012 | 0.9149 | 0.05% |
| ST | Env_PC1 | 1.0287 | 1.0287 | 1 | 8.332 | 0.8868 | 0.37285 | 3.90% |
| ST | Env_PC2 | 0.3956 | 0.3956 | 1 | 18.419 | 0.341 | 0.56635 | 1.50% |
| ST | PC1: Env_PC1 | 0.0033 | 0.0033 | 1 | 54.906 | 0.0029 | 0.95741 | 0.01% |
| ST | PC1: Env_PC2 | 0.0102 | 0.0102 | 1 | 38.691 | 0.0088 | 0.92578 | 0.04% |
| ST | PC2: Env_PC1 | 2.1537 | 2.1537 | 1 | 56.536 | 1.8565 | 0.17843 | 8.16% |
| ST | PC2: Env_PC2 | 0.7942 | 0.7942 | 1 | 75.67 | 0.6846 | 0.41062 | 3.01% |
| ST | PC3: Env_PC1 | 3.3162 | 3.3162 | 1 | 57.03 | 2.8586 | 0.09634 | 12.57% |
| ST | PC3: Env_PC2 | 7.4424 | 7.4424 | 1 | 20.54 | 6.4154 | 0.01955* | 28.20% |
| XT | PC1 | 42.65 | 42.65 | 1 | 78.071 | 0.3212 | 0.57248 | 1.52% |
| XT | PC2 | 375.75 | 375.75 | 1 | 85.809 | 2.8302 | 0.09614 | 13.36% |
| XT | PC3 | 1076.76 | 1076.76 | 1 | 62.016 | 8.1104 | 0.005962** | 38.28% |
| XT | Env_PC1 | 99.79 | 99.79 | 1 | 8.234 | 0.7517 | 0.41051 | 3.55% |
| XT | Env_PC2 | 543.58 | 543.58 | 1 | 13.346 | 4.0944 | 0.06353 | 19.33% |
| XT | PC1: Env_PC1 | 441.07 | 441.07 | 1 | 85.378 | 3.3223 | 0.07185 | 15.68% |
| XT | PC1: Env_PC2 | 132.95 | 132.95 | 1 | 87.053 | 1.0014 | 0.31974 | 4.73% |
| XT | PC2: Env_PC1 | 10.12 | 10.12 | 1 | 87.032 | 0.0762 | 0.78311 | 0.36% |
| XT | PC2: Env_PC2 | 23.45 | 23.45 | 1 | 86.988 | 0.1766 | 0.67534 | 0.83% |
| XT | PC3: Env_PC1 | 55.14 | 55.14 | 1 | 87.785 | 0.4153 | 0.52097 | 1.96% |
| XT | PC3: Env_PC2 | 11.46 | 11.46 | 1 | 70.897 | 0.0863 | 0.76975 | 0.41% |

*Significance: *, p < 0.05; **, p < 0.01; ***, p < 0.001.*

**Table S9** Summary results of niche equivalency and niche similarity tests. We set niches between ecotypes as inequivalent and dissimilar as alternative hypothesis.

|  | ***D*** | ***I*** |
| --- | --- | --- |
| Observed | 0.297 | 0.469 |
| Equivalency test *p*-value | 1.000 | 0.986 |
| Similarity test *p*-value | 0.974 | 0.939 |

**Table S10** Cases where gene flow counteracts genetic differentiation due to divergent selection, resulting in the genetic differentiation in neutral and adaptive loci.

| **Species** | **Gene Flow** | **Neutral loci Differentiation** | **Adaptive loci Differentiation** | | **Reference** |
| --- | --- | --- | --- | --- | --- |
| *Ipomoea* spp. | High | No | Yes | (Rifkin *et al.*, 2019) | |
| *Mimulus guttatus* | Moderate | No | Yes | (Aeschbacher *et al.*, 2017) | |
| *Timema cristinae* | Moderate | No | Yes | (Nosil *et al.*, 2008) | |
| *Dubautia* spp. | Variable | No | Yes | (Friar *et al.*, 2007) | |
| *Anthoxanthum odoratum* | High | Yes | Yes | (Freeland *et al.*, 2010) | |
| *Neochlamisus bebbianae* | Moderate | Yes | Yes | (Funk *et al.*, 2011) | |
| *Eperua falcate* | Restricted | Yes | Yes | (Brousseau *et al.*, 2015) | |
| *Tetranychus kanzawai* | Restricted | Yes | Yes | (Nishimura *et al.*, 2005) | |

References

**Aeschbacher S, Selby JP, Willis JH, Coop G. 2017.** Population-genomic inference of the strength and timing of selection against gene flow. *Proceedings of the National Academy of Sciences* **114**(27): 7061-7066.

**Berthet S, Thevenin J, Baratiny D, Demont-Caulet N, Debeaujon I, Bidzinski P, Leple J-C, Huis R, Hawkins S, Gomez L-D, et al. 2012.** Chapter 5 - Role of Plant Laccases in Lignin Polymerization. In: Jouanin L, Lapierre C eds. *Advances in Botanical Research*: Academic Press, 145-172.

**Bomal C, Bedon F, Caron S, Mansfield SD, Levasseur C, Cooke JEK, Blais S, Tremblay L, Morency M-J, Pavy N, et al. 2008.** Involvement of Pinus taeda MYB1 and MYB8 in phenylpropanoid metabolism and secondary cell wall biogenesis: a comparative in planta analysis. *Journal of Experimental Botany* **59**(14): 3925-3939.

**Brousseau L, Foll M, Scotti-Saintagne C, Scotti I. 2015.** Neutral and Adaptive Drivers of Microgeographic Genetic Divergence within Continuous Populations: The Case of the Neotropical Tree Eperua falcata (Aubl.). *PLOS ONE* **10**(3): e0121394.

**Burn JE, Hocart CH, Birch RJ, Cork AC, Williamson RE. 2002.** Functional Analysis of the Cellulose Synthase GenesCesA1, CesA2, and CesA3 in Arabidopsis. *Plant Physiology* **129**(2): 797-807.

**Ding W, Tu Z, Gong B, Deng Z, Liu Q, Gu Z, Yang C. 2024.** Unveiling Key Genes and Unique Transcription Factors Involved in Secondary Cell Wall Formation in Pinus taeda. *International Journal of Molecular Sciences* **25**(21): 11805.

**Durand M, Mainson D, Porcheron B, Maurousset L, Lemoine R, Pourtau N. 2018.** Carbon source–sink relationship in Arabidopsis thaliana: the role of sucrose transporters. *Planta* **247**(3): 587-611.

**Freeland JR, Biss P, Conrad KF, Silvertown J. 2010.** Selection pressures have caused genome-wide population differentiation of Anthoxanthum odoratum despite the potential for high gene flow. *J Evol Biol* **23**(4): 776-782.

**Friar EA, Cruse-Sanders JM, McGlaughlin ME. 2007.** Gene flow in Dubautia arborea and D. ciliolata: the roles of ecology and isolation by distance in maintaining species boundaries despite ongoing hybridization. *Mol Ecol* **16**(19): 4028-4038.

**Funk DJ, Egan SP, Nosil P. 2011.** Isolation by adaptation in Neochlamisus leaf beetles: host-related selection promotes neutral genomic divergence. *Mol Ecol* **20**(22): 4671-4682.

**Julius BT, Leach KA, Tran TM, Mertz RA, Braun DM. 2017.** Sugar Transporters in Plants: New Insights and Discoveries. *Plant and Cell Physiology* **58**(9): 1442-1460.

**Li QS, Liu C, Xin YX, Shen WX, Zhao WZ, Wang F, Cao ZY, Bai B, Xin PY. 2024.** <em>PINUS ARMANDII</em> GROWTH GENE IDENTIFICATION USING GENOME-WIDE ASSOCIATION STUDY APPROACHES. *Journal of Tropical Forest Science* **36**(1): 26-39.

**Nairn CJ, Lennon DM, Wood-Jones A, Nairn AV, Dean JFD. 2008.** Carbohydrate-related genes and cell wall biosynthesis in vascular tissues of loblolly pine (Pinus taeda)†. *Tree Physiology* **28**(7): 1099-1110.

**Nishimura S, Hinomoto N, Takafuji A. 2005.** Gene flow and spatio-temporal genetic variation among sympatric populations of Tetranychus kanzawai (Acari: Tetranychidae) occurring on different host plants, as estimated by microsatellite gene diversity. *Exp Appl Acarol* **35**(1-2): 59-71.

**Noman M, Siddique I, Saleem B, Ilyas S, Ali S, Khan MR. 2022.** In Silico Dissection and Expression Analysis of Sucrose Synthase Gene Family in Sugarcane. *Sugar Tech* **24**(6): 1766-1777.

**Nosil P, Egan SP, Funk DJ. 2008.** Heterogeneous genomic differentiation between walking-stick ecotypes: “Isolation by adaptation” and multiple roles for divergent selection. *Evolution* **62**(2): 316-336.

**Palle SR, Seeve CM, Eckert AJ, Cumbie WP, Goldfarb B, Loopstra CA. 2011.** Natural variation in expression of genes involved in xylem development in loblolly pine (Pinus taeda L.). *Tree Genetics & Genomes* **7**(1): 193-206.

**Rifkin JL, Castillo AS, Liao IT, Rausher MD. 2019.** Gene flow, divergent selection and resistance to introgression in two species of morning glories (Ipomoea). *Mol Ecol* **28**(7): 1709-1729.

**Shang X, Chai Q, Zhang Q, Jiang J, Zhang T, Guo W, Ruan Y-L. 2015.** Down-regulation of the cotton endo-1,4-β-glucanase gene KOR1 disrupts endosperm cellularization, delays embryo development, and reduces early seedling vigour. *Journal of Experimental Botany* **66**(11): 3071-3083.

**Zuo J, Niu Q-W, Nishizawa N, Wu Y, Kost B, Chua N-H. 2000.** KORRIGAN, an Arabidopsis Endo-1,4-β-Glucanase, Localizes to the Cell Plate by Polarized Targeting and Is Essential for Cytokinesis. *The Plant Cell* **12**(7): 1137-1152.
